# Regulation of Macrophage Motility by the Water Channel Aquaporin-1: Crucial Role of M0/M2 Phenotype Switch

**DOI:** 10.1371/journal.pone.0117398

**Published:** 2015-02-26

**Authors:** Donatienne Tyteca, Tomoya Nishino, Huguette Debaix, Patrick Van Der Smissen, Francisca N'Kuli, Delia Hoffmann, Yvette Cnops, Virginie Rabolli, Geert van Loo, Rudi Beyaert, François Huaux, Olivier Devuyst, Pierre J. Courtoy

**Affiliations:** 1 CELL Unit, de Duve Institute (DDUV), Université catholique de Louvain, Brussels, Belgium; 2 Pôle de Néphrologie (NEFR), Institut de recherche expérimentale et clinique (IREC), Université catholique de Louvain, Brussels, Belgium; 3 Louvain Centre for Toxicology and Applied Pharmacology (LTAP), Institut de recherche expérimentale et clinique (IREC), Université catholique de Louvain, Brussels, Belgium; 4 Division of Nephrology, Nagasaki University, Nagasaki, Japan; 5 Inflammation Research Center, VIB, Department of Biomedical Molecular Biology, University of Ghent, Ghent, Belgium; Medical University Vienna, Center for Brain Research, AUSTRIA

## Abstract

The water channel aquaporin-1 (AQP1) promotes migration of many cell types. Although AQP1 is expressed in macrophages, its potential role in macrophage motility, particularly in relation with phenotype polarization, remains unknown. We here addressed these issues in peritoneal macrophages isolated from AQP1-deficient mice, either undifferentiated (M0) or stimulated with LPS to orientate towards pro-inflammatory phenotype (classical macrophage activation; M1). In non-stimulated macrophages, ablation of AQP1 (like inhibition by HgCl_2_) increased by 2–3 fold spontaneous migration in a Src/PI3K/Rac-dependent manner. This correlated with cell elongation and formation of lamellipodia/ruffles, resulting in membrane lipid and F4/80 recruitment to the leading edge. This indicated that AQP1 normally suppresses migration of resting macrophages, as opposed to other cell types. Resting *Aqp1^-/-^* macrophages exhibited CD206 redistribution into ruffles and increased arginase activity like IL4/IL13 (alternative macrophage activation; M2), indicating a M0-M2 shift. In contrast, upon M1 orientation by LPS *in vitro* or peritoneal inflammation *in vivo*, migration of *Aqp1^-/-^* macrophages was reduced. Taken together, these data indicate that AQP1 oppositely regulates macrophage migration, depending on stimulation or not by LPS, and that macrophage phenotypic and migratory changes may be regulated independently of external cues.

## Introduction

Cell migration is a fundamental property for many physiological or pathological processes such as embryogenesis, angiogenesis, inflammation, stem cell repair and cancer. Individual cell migration generally involves a four-step process: membrane polarization, protrusion, cell traction and retraction. However, variations can be distinguished, based on differences in cell morphology, extent of adhesion to extracellular matrix and mechanics of leading edge protrusion, which appears to best define the mode of migration [1]. Thus, whereas many types of migrating cells display filopodia, lamellipodia are primarily found in highly motile cells. The Rho family GTPases, Rac1 and Cdc42, activate the actin nucleator Arp2/3 at the leading edge to polymerize actin and to form lamellipodia. PIP_3_, produced from PIP_2_ by PI3K phosphorylation, is also enriched in lamellipodia. The same type of migrating cells in 3D extracellular matrix can switch between different leading edge structures [1].

Changes in cell shape due to the rapid formation and retraction of lamellipodia and filopodia may be linked to changes in cell volume, which require water flow through specialized channels called aquaporins (AQPs), and indeed migration of some cells apparently depends on AQPs [2–5]. AQPs belong to a highly conserved family of membrane proteins called the major intrinsic proteins (MIPs) and 13 aquaporin isoforms have been identified so far in mammals (AQP0-12). Based on sequence similarity and substrate selectivity, they are grouped in three main subfamilies: (i) classical AQPs, considered as specific water channels (AQP0, AQP1, AQP2, AQP4, AQP5, AQP6 and AQP8); (ii) aquaglyceroporins, which also allow glycerol permeation (AQP3, AQP7, AQP9 and AQP10) [6]; and (iii) other AQPs localized intracellularly (AQP11, AQP12) [7]. The water transport function of AQPs facilitates urinary concentration, brain water homeostasis and cell migration [3], among others. The glycerol transport function of aquaglyceroporins plays roles in skin hydration, wound healing, cellular energy metabolism [8] and in macrophage immune functions [2]. Gating of AQPs has been recently described and factors like pH, pressure, temperature and membrane tension have been reported to affect the gating/opening of AQPs [9–15].

The first AQP, identified as CHIP28 in erythrocytes by Agre and colleagues [16], was renamed AQP1. In the kidney, AQP1 mediates osmotically driven water transport across epithelial and endothelial cells (reviewed by [17]). Outside the kidney, AQP1 is abundantly expressed in endothelial cells lining non-fenestrated capillaries of several organs outside the central nervous system [18]. AQP1 is known to facilitate migration of several cell types: (i) stable transfection of CHO cells with AQP1 accelerates migration [3]; (ii) in proximal tubule cells cultured from AQP1 KO mice, migration is decreased as compared to WT cells [4]; and (iii) in AQP1 deficient aortic endothelial cells, cell migration is also greatly impaired and leads to abnormal vessel formation [3]. In human melanoma cells, AQP1 co-immunoprecipitates with several transporters involved in migration [19] and contributes to cell migration through Lin7/beta-catenin interaction [20]. Thus, AQP1 accelerates migration of endothelial, epithelial and melanoma cells, suggesting a role in vasculogenesis and cancer spread.

Endothelial and epithelial cells, which have elaborate cytoskeleton, usually show collective slow migration. In contrast, macrophages have a poorly-organized actin cytoskeleton, move as individuals, and play crucial roles in immunity, since they sense and move to the site of infection or injury (migration) and engulf microorganisms, foreign particles and apoptotic bodies (phagocytosis) [21,22]. Moreover, macrophages are a heterogeneous cell population, able to switch phenotype in response to environmental cues. The two main phenotypes are (i) classically activated macrophages (M1), which exhibit pro-inflammatory, anti-tumor and anti-microbial properties; and (ii) alternatively activated macrophages (M2), which show anti-inflammatory activity and are instead involved in tissue remodelling, healing and repair [22–24]. *In vitro*, these phenotypes can be readily induced from undifferentiated macrophages (M0) by cytokines and other stimuli: (i) interferon-γ (IFNγ) and/or lipopolysaccharide (LPS) orient to M1 phenotype; (ii) combination of IL4/IL13 is the most common way to generate M2 macrophages [22,24]. M1 macrophages produce nitric oxide (NO) and reactive oxygen species (ROS), making them cytotoxic. The main characteristics of M2 macrophages are instead (i) production of growth factors; (ii) expression of high levels of arginase; and (iii) up-regulation of several non-opsonic receptors such as mannose receptor (CD206) [25]. M1 and M2 macrophages also exhibit different features with respect to cell shape, cytoskeletal organization and migration [26–28].

Whereas AQP1 accelerates migration of endothelial and epithelial cells, its role in macrophage migration in the context of phenotype polarization is unknown. Using peritoneal macrophages isolated from AQP1-deficient mice that we either left undifferentiated (M0) or orientated to M1 phenotype, we found that AQP1 ablation strongly impacts on macrophage morphology, cytoskeletal organization, membrane polarization and cell migration. We further demonstrate an opposite effect of AQP1 on macrophage migration, depending on external stimuli. Altogether, our results indicate a differential role of AQP1 in macrophage migration, depending on resting *vs* LPS-stimulated state.

## Material and Methods

### Animals and macrophage isolation

AQP1 KO mice were kindly provided by Dr. Alan S. Verkman [29,30]. We used C57BL6/J gender-matched WT or *Aqp1* ^*-/-*^ littermates aged 8–12 weeks. The mice had free access to appropriate standard diet (Carfil Quality, Oud-Turnhout, Belgium). All procedures were performed in accordance with National Institutes of Health guidelines for the care and use of laboratory animals and with the approval of the Committee for Animal Rights of the UCL Medical School (Brussels, Belgium). Animals were killed by subcutaneous injection of 24mg sodium pentobarbital (Certa). The peritoneal cavity was first lavaged with 10ml sterile NaCl (0.9%). Peritoneal lavage fluids were collected from wild-type or *Aqp1* ^-/-^ mice, pooled and centrifuged (280*g*, 10min, 4°C). Cell pellets were suspended in DMEM/F12 medium with 10% fetal bovine serum (hereafter called serum; both from Invitrogen) and macrophages were allowed to adhere in culture plates for 2h. Non-adherent cells were removed by washing and the adherent cells (>99% macrophages) were maintained for 24h in 10% serum-containing medium.

### Macrophage phenotype induction and treatments

To induce macrophage phenotype switch *in vitro*, cells were further incubated for 24h in the following media: (i) serum-free medium or 10% serum-containing medium, to maintain undifferentiated M0 state; (ii) serum-containing medium supplemented with 1μg/ml LPS alone or 100ng/ml LPS combined with 100ng/ml recombinant mouse IFNγ to orient towards M1 differentiation; or (iii) serum-containing medium supplemented with 20ng/ml IL4 combined with 10ng/ml IL13, to induce M2 differentiation. When appropriate, medium was supplemented with SU6656 (3μM, Src kinases inhibitor), wortmannin (100nM, irreversible PI3kinase inhibitor) or NSC23766 (100μM, Rac inhibitor); wortmannin was removed after 1h and replaced by fresh medium. For treatment with HgCl_2_, cells were preincubated in serum-free medium supplemented with 1μM HgCl_2_ for 30min, then incubated for 5h in serum-containing medium in the continued presence of HgCl_2_, after which the compound was removed.

### RT-qPCR

Total RNA from macrophages was extracted with RNAqueous^R^-Micro kit (Ambion) following the manufacturer’s protocol and reverse-transcribed into cDNA with iScript cDNA Synthesis Kit (Bio-Rad). Changes in target genes mRNA levels were determined by relative RT-qPCR with a CFX96 Real-Time PCR Detection System (Bio-Rad) using iQ SYBR Green Supermix (Bio-Rad) detection of single PCR product accumulation. RT-qPCR analyses were performed in duplicate with 100nM of both sense and anti-sense primers in a final volume of 20μl using iQ SYBR Green Supermix (Bio-Rad). Specific primers were designed using Primer3 (S1 Table). PCR conditions were 95°C for 3min followed by 40 cycles of 15sec at 95°C, 30sec at 60°C. The PCR products were sequenced with the BigDye terminator kit (Perkin Elmer Applied Biosystems). The multiScreen SEQ_384_ Filter Plate (Millipore) and Sephadex G-50 DNA Grade Fine (Amersham Biosciences) dye terminator removal were used to purify sequence reactions before analysis on an ABI3100 capillary sequencer (Perkin Elmer Applied Biosystems). The efficiency of each set of primers was determined by dilution curves (S1 Table). The relative changes in target gene/GAPDH mRNA ratio were determined by the formula: 2 ^∆∆ct^ [31] (The MIQUE Guidelines, 2009).

### Flow Cytometry Analysis

Macrophages were resuspended in Hanks' medium with 3% decomplemented serum and 10mM NaN_3_. Fc receptors were blocked with anti-CD16/32 (clone 2.4G2; BD Biosciences). Cells were co-labelled for F4/80 (clone BM8; eBioscience) and CD11b (clone M1/70; BD Biosciences). Samples were fixed by 1.2% formaldehyde in PBS for 1h and analyzed on a FACSCalibur with data processing using CellQuest software (both from BD Biosciences). Gating was set according to side with forward scatter to exclude dead cells.

### Phagocytosis determination and (immuno)fluorescence

Macrophages were seeded in serum-containing medium on collagen I-coated coverslips at 20.000cells/cm^2^, maintained for 24h in serum-containing medium and appropriate phenotypes were induced (see above, section “Macrophage phenotype induction and treatments”). Cells were then processed for immunofluorescence as described by [32]. The following antibodies were used: rat monoclonal antibody against mouse F4/80 (Abd Serotec) and rabbit polyclonal antibodies against mannose receptor (CD206; Abcam). To test for macrophage purity and to measure phagocytosis, macrophages were allowed to phagocytose 1μm-green latex beads (Sigma) in medium supplemented with 1% bovine serum albumin (BSA) for 1h at 37°C and washed 5 times with PBS at 4°C. This washing procedure allows to remove >90% of the beads bound at 4°C [33]. Cells were then fixed/permeabilized and (immuno)labelled. Preparations were examined with a Zeiss LSM510 confocal microscope using a plan-Apochromat 63x NA 1.4 oil immersion objective.

### Migration assays

#### Wound healing recolonization assay

Cells were seeded in serum-containing medium in 12-well plastic plates at 100.000cells/cm^2^ and appropriate phenotypes were induced for 24h (see above). After stripping a linear path with a razor-blade, non-adherent cells were washed away and adherent cells allowed to migrate into the wound at 37°C for 24h in the appropriate migration medium. Except otherwise stated (see S2 Fig.), migration medium contained 10% serum, either alone or supplemented with 1μg/ml LPS or pharmacological agents, as indicated in the figure legends. After 5 washes with PBS, cells were formaldehyde-fixed, stained with 0.5% crystal violet and washed (adapted from [34]). The number of cells that had colonized the wound was determined in at least 10 microscopic fields per condition using a 20x objective and expressed as percentage of cells present in a same surface area far from the stripping band.

#### De novo colonization in plastic IBIDI chamber

Cells were seeded as above in serum-containing medium in IBIDI chambers equipped with a culture insert preventing local attachment (IBIDI, Proxylab). After 24h, insert was removed and cells allowed to migrate in serum-free medium into the exposed area for 24h, washed, fixed, stained and counted as above.

#### Transwell migration assay

Cells were seeded in serum-containing medium at 200.000cells/polyethylene terephthalate insert with a 8μm-pore size in 24-well plates (BD BioCoat). Medium in the upper chamber was then replaced by serum-free medium and cells were allowed to migrate towards serum-free medium for 24h. At the end, the filter was rinsed 5 times with PBS at the upper and lower chambers, fixed and stained as above. Cells remaining in the upper part of the insert were wiped with a cotton-tipped swab and the filter was mounted upside-down on a glass slide. The number of cells that had migrated to the lower surface was determined in at least 10 microscopic fields per condition.

### Labelling with BODIPY-sphingomyelin and vital imaging.

Cells were seeded at 100.000cells/cm^2^ in IBIDI chambers with culture insert (IBIDI, Proxylab). After 24h, insert was removed and cells allowed to migrate in serum-containing medium at 37°C into the cell-free area. Cells were then labelled with 1μM BODIPY-sphingomyelin (BODIPY-SM; Invitrogen) and 10μg/ml FM4–64 (red; Invitrogen) in medium containing equimolar defatted bovine serum albumin (DF-BSA) at 4°C for 15min [35–38]. After washing, cells were immediately analyzed at room temperature with the Zeiss LSM510 confocal microscope using a plan-Apochromat 63x NA 1.4 oil immersion objective.

### Scanning electron microscopy.

Macrophages were seeded on collagen I-coated-coverslips at 20.000cells/cm^2^ and maintained for 24h in serum-containing medium. After phenotype induction for 24h (see above), cells were extensively washed with PBS, rinsed twice in 0.14M cacodylate buffer, pH 7.4, then fixed with glutaraldehyde at room temperature by carefully increasing fixative concentrations (0.1, 0.5 and 1% (v/v) in 0.1M cacodylate buffer for 30min each), followed by 2% glutaraldehyde at 4°C overnight. The next day, samples were extensively washed in 0.1M cacodylate buffer and post-fixed with 1% (w/v) OsO_4_ at 4°C for 2h. Samples were dehydrated in graded ethanol series and critical-point dried (CPD 0.20, Balzers Union). A 10-nm gold film was sputter-coated and specimens were observed in a CM12 electron microscope at 80kV with the use of the secondary electron detector (Philips).

Determination of arginase activity. Arginase activity was measured in cell lysates, as described [39]. At 24h after stimulation (see above), cultured macrophages were lysed with 80μl 0.1% Triton X-100. After 30min of shaking, arginase was activated by addition of 100μl Tris-HCl (25mM) and 35μl MnCl_2_ (10mM) and incubation at 56°C for 10min, then L-arginine hydrolysis was allowed by incubation with 100μl L-arginine (0.5M, pH 9.7) at 37°C for 1h. The reaction was stopped with 800μl H_2_SO_4_ (96%)/H_3_PO_4_ (85%)/H_2_O (1:3:7). The produced urea was quantified at 540nm after addition of 40μl α-isonitrosopropiophenone (9%, dissolved in 100% ethanol), followed by heating at 100°C for 20min. One unit of enzyme is defined as the amount that catalyzes the formation of 1μmol urea/min.

Determination of NO production. NO production was monitored by assessing nitrite levels in the extracellular medium of cells stimulated for 24h (see above) using Griess reagent. Briefly, 100μl of Griess reagent (5% H_3_PO_4_, 1% sulfanilamide, 0.1% N-1-naphtyl-ethylendiamine) was added to 50μl cell-free supernatants and absorbance was measured at 540nm. Biochemical analyses were performed in triplicate, by reference to a standard curve of increasing sodium nitrite concentrations.

### Western blotting.

Macrophages were seeded at 6.10^6^cells/20cm^2^-non coated Petri dish and maintained for 24h in serum-containing medium. After phenotype induction (see above), proteins of cell lysates were resolved by SDS-PAGE (4–15% acrylamide, Bio-Rad) and electro-blotted onto PVDF membranes (NEN). After saturation for at least 2h in blotting buffer (50mM Tris, pH 8.0, 90mM NaCl, 2mM CaCl_2_, 0.15% [w/v] Tween 20 and 5% [w/v] milk powder), membranes were incubated overnight at 4°C with rabbit polyclonal primary antibodies (AQP1 [Chemicon International], total p38 and p38-pT180/Y182 [all from Cell Signaling]) or mouse IgG1 anti-iNOS (BD Transduction Laboratories) or anti-IκBα (Santa Cruz Biotechnology Inc) and anti-pIκBα (Cell Signaling). After 5 washes, blots were incubated at room temperature with the appropriate HRP-conjugated secondary antibodies in 2% BSA-containing buffer for 1h, washed again 5 times, transferred to PBS and revealed by chemoluminescence (ECL kit, Perkin Elmer) using Kodak X-omat blue films and scanned at high resolution (Agfa SNAPscan 600).

### Catheter model.

The inflammatory response *in vivo* was investigated using a well-established mouse model of acute peritonitis [40]. Acute peritonitis was generated by insertion of a peritoneal catheter as described previously [41]. At day 0, mice were anesthetized with ketamine (100mg/kg subcutaneously; Merial) and xylazine (10mg/kg subcutaneously; Bayer). A silicone catheter (Terumo) was implanted into the peritoneal cavity without aseptic precautions and subcutaneously tunneled to the neck. A daily infusion of 2ml of dialysate (3.86% Dianeal; Baxter) was performed for 6d. In addition, animals were intraperitoneally administered Staphylococcus epidermidis (10^7^/ml colony-forming units, diluted in 3.86% dialysate) at day 1 and day 3. On day 7, mice were submitted to a 2h-PD exchange to measure permeability parameters followed by tissue sampling.

### Morphometry.

Colocalization between green phalloidin and red F4/80 was estimated by yellow signal. CD206 recruitment into membrane cellular extensions was defined as cellular extensions labelled by F4/80 showing green dots of CD206 at their ends. Lipid polarization was measured on macrophages double-labelled for BODIPY-SM (green) and the artificial lipid dye, FM4-64 (red), by recording a line intensity profile from the leading to the trailing edges of the cells. The ratio of green (BODIPY-SM) / red (FM4-64) was determined at each edge and results were expressed as ratio at leading *vs* trailing edge. Morphometric measurement of cell elongation was performed with the Axiovision software. Macrophages labelled by phalloidin were manually surrounded and thresholding of the image was performed. From this image, the smallest and largest diameters of each cell were automatically determined by constructing the Ferret box (standard measuring function in AxioVision) and the elongation factor for each cell (largest/smallest diameter ratio) was calculated.

### Statistical analyses.

Values are presented as means±SEM. The significance of differences was tested by Student’s t-test (two-group comparisons) or by one way ANOVA followed by Newman-Keuls test (multiple comparisons), as appropriate. *, p<0.05; **, p<0.01; ***, p<0.001; NS, not significant.

## Results

### Ablation or chemical inhibition of AQP1 increases migration of non-stimulated macrophages

To examine whether AQP1 could play a role in macrophage migration, we used freshly isolated peritoneal macrophages from *Aqp1* ^*-/-*^ mice. Macrophages were retrieved in similar abundance from *Aqp1* ^*+/+*^ and *Aqp1* ^*-/-*^ mice, as shown by FACS based on immunolabeling for the cell surface glycoprotein F4/80 [42] and for CD11b (S1A Fig.). By confocal microscopy, *Aqp1* ^*+/+*^ and *Aqp1* ^*-/-*^ macrophages, identified by phagocytosis of latex beads and F4/80 expression (S1B Fig.), represented >99% of collected cells. At mRNA level, AQP1 was among the most abundant isoforms in WT macrophages (S2 Table). Absence of AQP1 expression in KO macrophages was verified by western blot (S1C Fig.).

**Fig 1:**
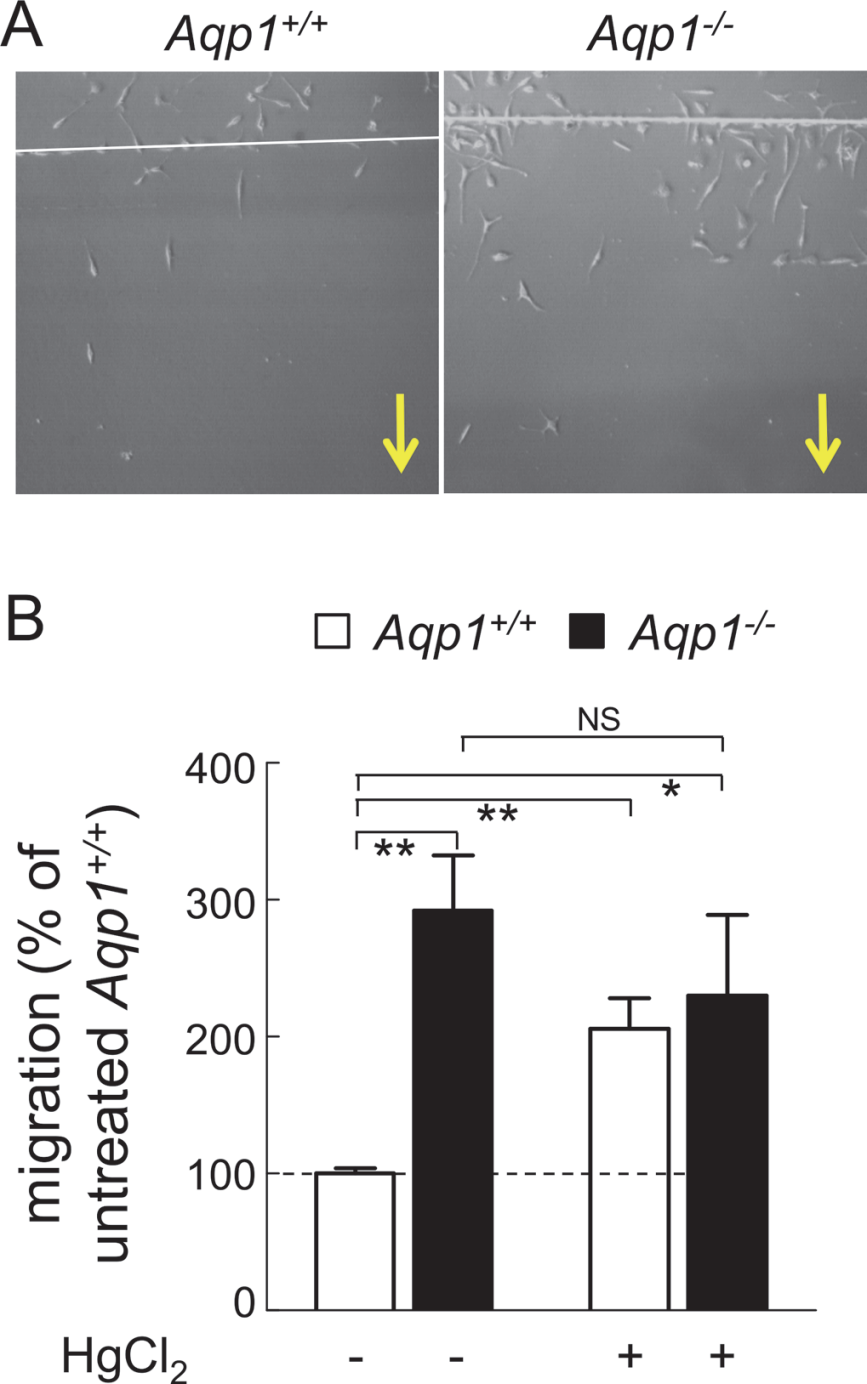
Motility of non-stimulated macrophages is increased by AQP1 ablation and by HgCl_2_. *Aqp1* ^*+/+*^ (open bars) and *Aqp1* ^*-/-*^ (filled bars) macrophages were maintained in 10% serum or preincubated with 1μM HgCl_2_ for 30min prior to linear scraping for wound healing assay. Cells were allowed to recolonize the wound in serum-containing medium for 5h, in the continued presence of HgCl_2_ if appropriate, then HgCl_2_ was removed and migration allowed to continue for 20 additional hours. After washing, cells were stained and macrophages that had colonized the wound were counted, normalized to the population introduced in the well, then expressed by reference to untreated *Aqp1* ^*+/+*^ macrophages. **(A) Representative images.** Yellow arrows indicate direction of migration. **(B) Quantification.** The number of macrophages that had colonized the wound was determined in at least 10 microscopic fields per condition using a 20x objective and expressed as percentage of cells present in a same surface area far from the stripping band. This ratio was ∼10% for untreated *Aqp1* ^*+/+*^ cells (1^st^ column). All results were then expressed by reference to untreated *Aqp1* ^*+/+*^ cells. Values are means±SEM of 4–6 experiments with 1–3 dishes each. NS, not significant; *, p<0.05; **, p<0.01. For wound without serum and complementary IBIDI and transwell assays, see S2 Fig.

Spontaneous migration of *Aqp1* ^*-/-*^ macrophages was first tested by wound healing assay in medium with serum only, to maintain a non-differentiated M0 phenotype. Motility was constitutively increased by ∼3-fold upon AQP1 ablation (Fig. 1A; quantification at Fig. 1B). Stimulation of motility was also observed in serum-free medium in three different assays: (i) recolonization by wound healing assay (S2A Fig.); (ii) *de novo* colonization in plastic IBIDI chambers (S2B Fig.); and (iii) Transwell migration assay (S2C Fig.). Taken together, these data demonstrate a constitutive stimulation of migration in non-differentiated *Aqp1* ^*-/-*^ macrophages, indicating a normal repression of migration by AQP1 in WT macrophages.

To exclude that stimulation of non-differentiated *Aqp1* ^*-/-*^ macrophages resulted from a compensatory overexpression of other aquaporins, mRNA levels of several aquaporins and aquaglyceroporins were determined by RT-qPCR. No important differences could be found between *Aqp1* ^*-/-*^ and *Aqp1* ^+/+^ macrophages; only AQP7 mRNA levels were moderately increased in *Aqp1* ^*-/-*^ macrophages (S2 Table). We therefore further investigated migration of peritoneal macrophages similarly collected from *Aqp7* ^-/-^ mice. As shown by S3 Fig., migration of *Aqp7* ^*-/-*^ and *Aqp7* ^*+/+*^ macrophages was similar, suggesting that the strong stimulation of spontaneous migration we observed in *Aqp1* ^*-/-*^ macrophages was not due to AQP7 overexpression.

As an alternative approach to evaluate the effect of AQP1 on macrophage motility, macrophages were treated with 1μM HgCl_2_, as AQP1 blocker [43]. Up to 10μM, HgCl_2_ was not toxic for macrophages and did not induce apoptosis [44]. This SH-binding agent stimulated by ∼2-fold the spontaneous migration of *Aqp1* ^*+/+*^ macrophages but did not further promote motility of *Aqp1* ^*-/-*^ macrophages (Fig. 1B, right), suggesting specificity of blocking. These results indicated that genetic ablation or acute chemical blockade of AQP1 were equivalent to stimulate the spontaneous migration of non-differentiated macrophages.

### Increased migration of non-stimulated macrophages depends on the Src/PI3K/Rac pathway

**Fig 2:**
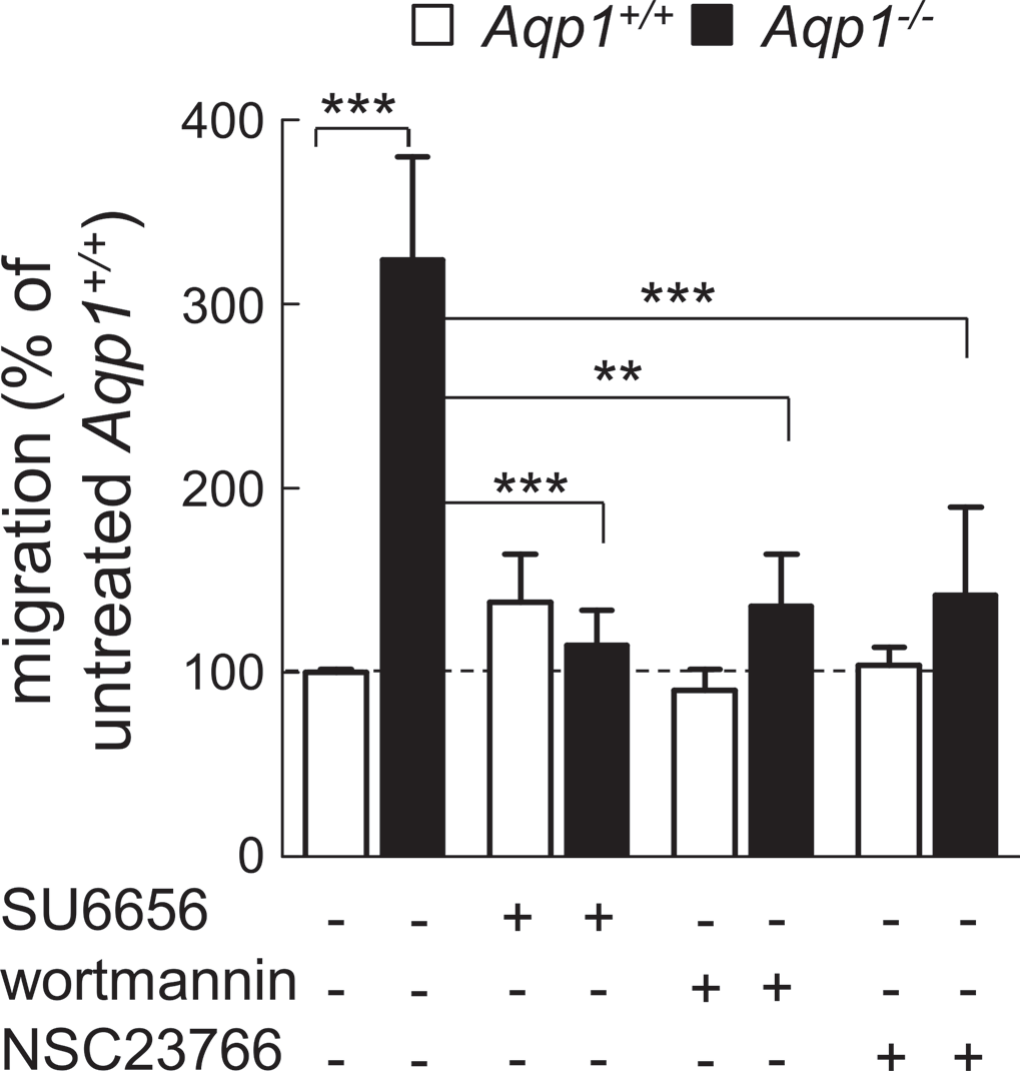
Increased macrophage motility upon AQP1 ablation depends on Src/PI3K/Rac signalling. Motility of *Aqp1* ^*+/+*^ and *Aqp1* ^-/-^ macrophages was tested by wound healing assay as at Fig. 1 in serum-containing medium supplemented or not with inhibitors of Src kinases (3μM SU6656 for 24h), PI3K (100nM wortmannin for 1h, then chase in fresh medium) or Rac1 (100μM NSC23766 for 24h). Quantification was performed as at Fig. 1B and values are means±SEM of 4 separate experiments with 1–2 dishes each. **, p<0.01; ***, p<0.001.

Since Rac function is crucial for cell migration [45], we next tested whether stimulation of cell migration by AQP1 ablation depends on the Src/PI3K/Rac signalling pathway. Src, PI3K and Rac were respectively inhibited by SU6656, wortmannin and NSC23766 [46,47]. Whereas migration of WT macrophages was insensitive to these three inhibitors, stimulation of migration induced by AQP1 ablation was fully abrogated (Fig. 2), consistent with the hypothesis of constitutive activation of the Src/PI3K/Rac signaling pathway.

### Ablation of AQP1 spontaneously induces macrophage elongation, axial polarization and membrane lipid orientation to the leading edge

**Fig 3:**
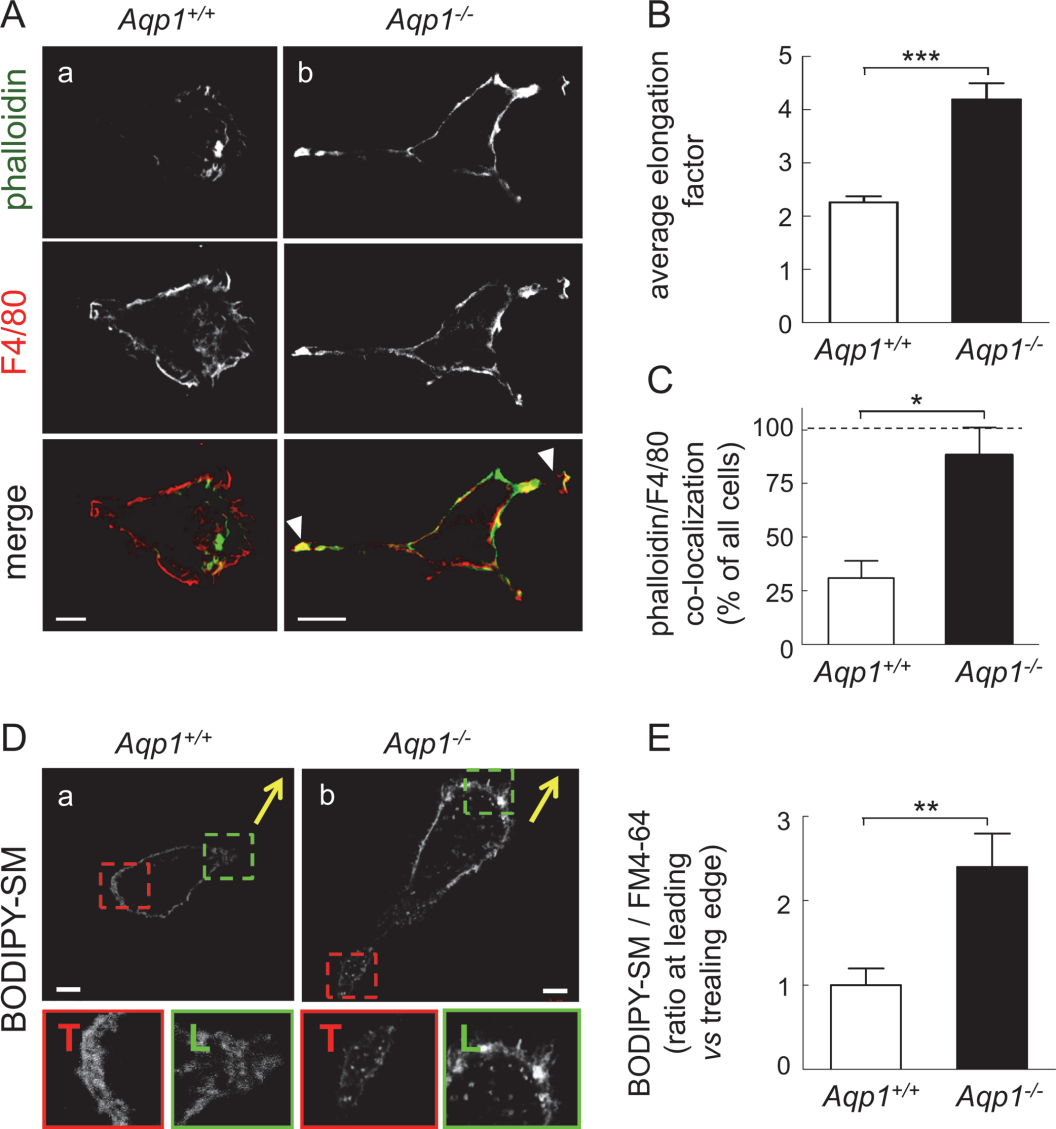
AQP1 ablation induces macrophage elongation and favors axial and membrane lipid polarity. **(A-C) Macrophage elongation and axial polarity. (A) Representative confocal images.** *Aqp1* ^*+/+*^ and *Aqp1* ^-/-^ macrophages were maintained for 24h in serum-containing medium then immunolabeled for F4/80 (macrophage plasma membrane; red) by reference to F-actin (phalloidin; green). Confocal images shown are representative of 3 experiments and general views are provided at S4 Fig. Scale bars, 5μm. Notice elongation of *Aqp1* ^*-/-*^ macrophages (quantified at B) and the usual spontaneous occurrence of large cellular extensions supported by thick actin filaments and concentrating the F4/80 marker (arrowheads; quantification at C). **(B) Quantification of cell elongation** by the ratio of the length of the long axis to the short axis (see also Material and Methods section) on 104 *Aqp1* ^*+/+*^ and 56 *Aqp1* ^-/-^ macrophages (pooled from 2 independent experiments). Data are means±SEM. **(C) Quantification of colocalization between F4/80 and phalloidin at cellular extensions.** Analysis was performed on 28 *Aqp1* ^*+/+*^ and 20 *Aqp1* ^*-/-*^ macrophages, pooled from 2 independent experiments. **(D,E) Membrane lipid polarity. (D) Representative confocal imaging.** *Aqp1* ^*+/+*^ and *Aqp1* ^*-/-*^ macrophages were plated with serum-containing medium in IBIDI chambers under locking the cell-free area, then all chambers were reincubated in fresh serum-containing medium after removal of the lock to allow cell migration for 24h. After this interval, monolayers were briefly labelled with a fluorescent analog of sphingomyelin (BODIPY-SM) at 4°C to prevent internalization, and cells that had migrated into the originally cell-free area were immediately analysed by vital confocal imaging for the localization of BODIPY-SM at the leading (L) or trailing edge (T), as better seen as enlargements in the green (L) and red (T) insets. Yellow arrows indicate directionality of migration. Scale bars, 5μm. **(E) Quantification of macrophages showing BODIPY-SM polarization.** Lipid polarization was measured on macrophages double-labelled for BODIPY-SM (green; Fig. 3D) and the artificial lipid dye, FM4-64 (red; not shown at Fig. 3D). The ratio of BODIPY-SM/FM4-64 fluorescence was determined at each edge and results were expressed as ratio at leading *vs* trailing edge (see also Material and Methods section). Analysis was performed on 17 *Aqp1* ^*+/+*^ and 16 *Aqp1* ^*-/-*^ macrophages that had colonized the unlocked area (pooled from 2 independent experiments). *, p<0.05; **, p<0.01; ***, p<0.001.

In order to address the underlying mechanism linking AQP1 and cell migration, we tested by confocal microscopy whether AQP1 ablation could influence overall cell shape as well as microfilament and membrane polarization, based on the comparison of F-actin labelling with that of F4/80 at the cell surface. *Aqp1* ^*-/-*^ macrophages were clearly more elongated than *Aqp1* ^*+/+*^ macrophages (Fig. 3A; for general views, see S4 Fig.). This was confirmed by quantification of the cell elongation factor (Fig. 3B); in contrast, projected cell area was unchanged (data not shown). Moreover, whereas phalloidin and F4/80 labelling were largely segregated in WT cells (Fig. 3Aa), large cellular extensions supported by thick actin filaments and concentrating the F4/80 marker (arrowheads at Fig. 3Ab, merge) were spontaneously observed in *Aqp1* ^*-/-*^ macrophages (quantification at Fig. 3C). Taken together, these data indicated that AQP1 ablation has a strong impact on cell shape and could favour axial polarization.

This was confirmed at the membrane lipid level. Plasma membrane lipid organization was analyzed in migrating cells in IBIDI chambers, using a fluorescent analog of sphingomyelin (BODIPY-SM) that readily inserts in the outer plasma membrane leaflet [35,36]. Whereas BODIPY-SM labelling was similar at the trailing (T) and the leading (L) edges of WT cells (insets at Fig. 3Da), BODIPY-SM concentration at the leading edge was clearly visible in *Aqp1* ^*-/-*^ macrophages (Fig. 3Db and quantification at Fig. 3E).

### Ablation of AQP1 induces M0 to M2 macrophage differentiation

We then turned our attention to the comparison of AQP1 ablation with the M0 to M1 and M0 to M2 phenotype switch. Macrophages are plastic cells that exhibit distinct functional characteristics with respect to cell morphology, cytoskeletal organization and migration, depending on a M0 to M1 or M0 to M2 switch. Among these features for M2 are: cell elongation [27], formation of lamellipodia [48,49], and increased migration [26]. We therefore asked whether constitutive elongation and higher migration of macrophages upon AQP1 ablation were the equivalent to a M0 to M2 phenotypic transition.

**Fig 4:**
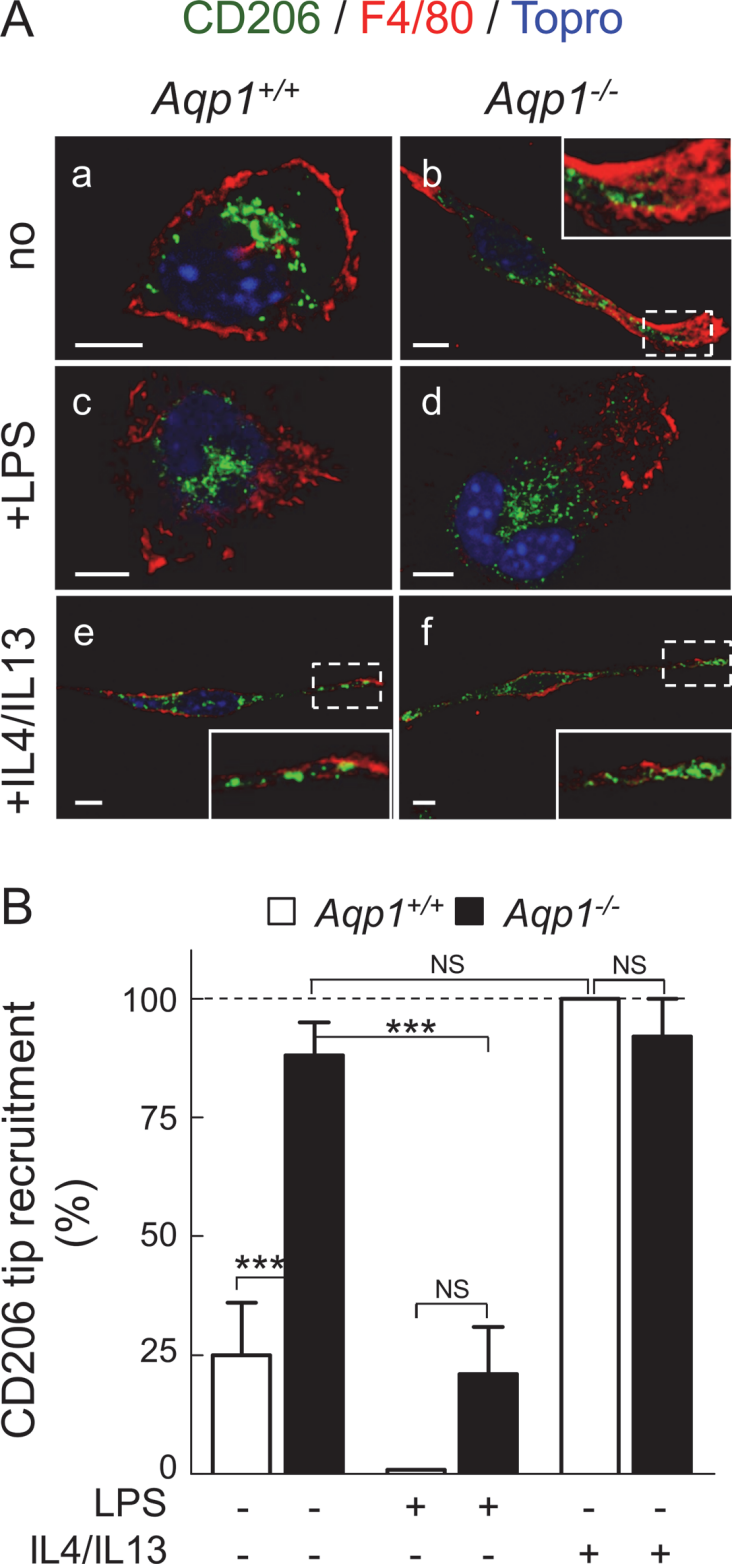
AQP1 ablation mimicks M2 functional differentiation induced in WT macrophages by IL4/IL13: A- CD206 redistribution into ruffles. (A) Confocal images. *Aqp1* ^*+/+*^ and *Aqp1* ^-/-^ macrophages were maintained for 24h in serum-containing medium without supplements (to keep macrophages in undifferentiated M0 state; upper row), or with LPS (to orient towards M1 phenotype; middle row) or IL4/IL13 (to orient towards M2 phenotype; lower row). Cells were fixed/permeabilized and double-immunolabelled for CD206 (green) and F4/80 (red) by reference to nuclei (Topro-3; blue). Representative of 2 independent experiments. Insets of M0 (*Aqp1* ^*-/-*^; panel b) and M2 macrophages (*Aqp1* ^*+/+*^ and *Aqp1* ^-/-^; panels e and f) show strong recruitment of CD206 (green) into ruffles, contrasting with preferential perinuclear localization in WT M0 and M1 macrophages (panels a and c). Scale bars, 5μm. **(B) Morphometry: redistribution of CD206 in ruffles.** From left to right, analysis was performed on 54, 35, 30, 31, 10 and 7 cells, pooled from 2 independent experiments. NS, not significantly different; ***, p<0.001.

To test for this hypothesis, we first evaluated by confocal microscopy the distribution of CD206, a M2 marker [25], in macrophages either non-stimulated or stimulated by LPS to orient to M1 phenotype or IL4/IL13 to orient instead towards M2 phenotype. In WT macrophages, CD206 was perinuclear in M0 and M1 states (Fig. 4Aa,c), but translocated to the plasma membrane at cellular extensions concentrating the F4/80 marker upon IL4/IL13 (Fig. 4Ae). In *Aqp1* ^*-/-*^ macrophages, CD206 was spontaneously recruited to similar extensions irrespective of stimulation or not by IL4/IL13 (Fig. 4Ab,f; quantification at Fig. 4B), thus mimicking M2 functional differentiation induced by IL4/IL13 in WT cells. Constitutive CD206 redistribution to ruffles provided the first line of evidence that AQP1 ablation can mimic M2 functional differentiation.

**Fig 5:**
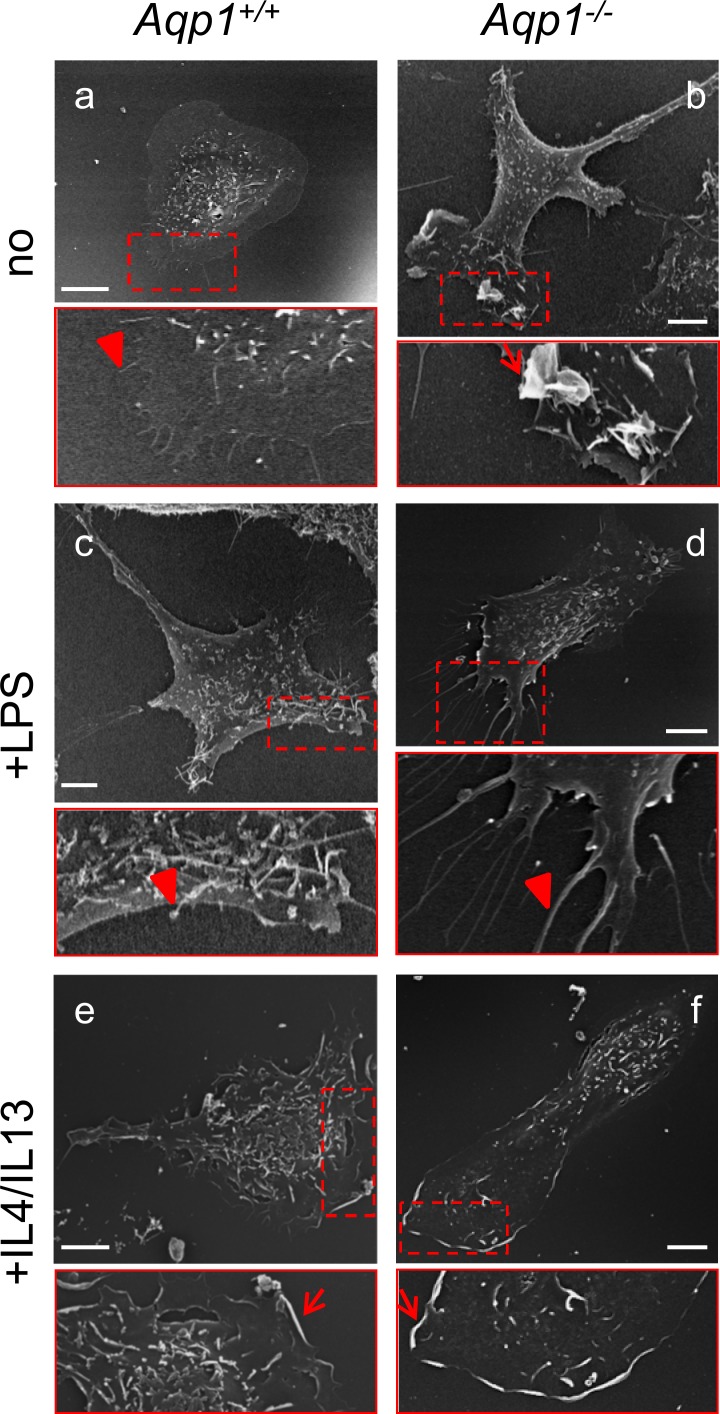
AQP1 ablation mimicks M2 functional differentiation induced in WT macrophages by IL4/IL13: B- constitutive induction of lamellipodia. *Aqp1* ^*+/+*^ and *Aqp1* ^-/-^ macrophages were maintained for 24h in serum-containing medium without supplements (upper row), with LPS (middle row) or IL4/IL13 (lower row) as at Fig. 4, then analysed by scanning electron microscopy. Scale bars, 5μm. Images are representative of 3 independent experiments at a,b, and 1–2 experiments at c-f. Insets focus on cell extremities and show mostly filopodia (arrowheads) at a,c,d but lamellipodia (arrows) at b,e,f. Notice coupling of cell elongation and lamellipodia/ruffles in *Aqp1* ^*-/-*^ macrophages (b), irrespective of IL4/IL13 treatment (e).

As a second line of evidence, we looked by scanning electron microscopy for lamellipodia, a hallmark of M2 macrophages [48,49], in *Aqp1* ^*-/-*^ macrophages. Lamellipodia were essentially absent in unstimulated WT cells (Fig. 5A) but induced by IL4/IL13 (Fig. 5E). The non-stimulated yet elongated *Aqp1* ^*-/-*^ macrophages clearly showed cellular extensions (Fig. 5B) concentrating lamellipodia at one end (inset at Fig. 5B). This contrasted with filopodia induced by LPS treatment in both WT and *Aqp1* ^*-/-*^ macrophages (insets at Fig. 5C,D), as expected [50]. Thus, constitutive formation of lamellipodia (but not of filopodia) in non-stimulated *Aqp1* ^*-/-*^ macrophages provides the second evidence that AQP1 ablation can mimic M2 functional differentiation.

**Fig 6:**
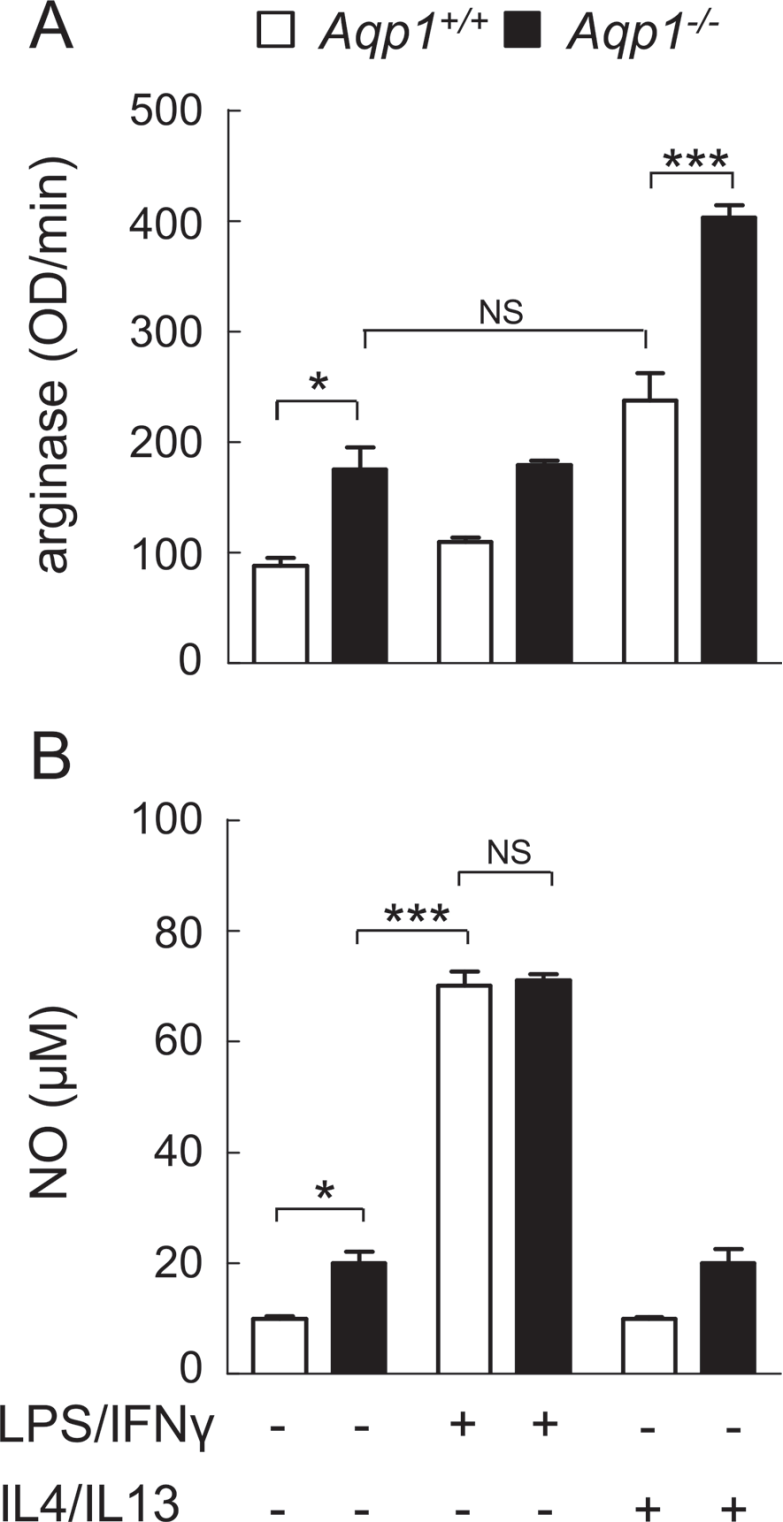
AQP1 ablation mimicks M2 functional differentiation induced in WT macrophages by IL4/IL13: C- constitutive increase of arginase activity. *Aqp1* ^*+/+*^ and *Aqp1* ^-/-^ macrophages were incubated with serum-containing medium alone or supplemented with LPS/IFNγ or with IL4/IL13, then assessed by spectrophotometry for arginase activity (M2 differentiation; A) or NO (M1 differentiation; B). Notice that AQP1 ablation constitutively increases arginase activity, to a similar level as IL4/IL13. NS, not significantly different; *, p<0.05; ***, p<0.001.

We finally quantified arginase activity, a M2 marker, *vs* NO production, a M1 marker [22,24,51]. It was first verified in WT macrophages that arginase activity and NO production can be respectively induced by IL4/IL13 and LPS/INFγ. As expected, IL4/IL13, but not LPS/INFγ treatment, stimulated arginase activity (compare open columns at Fig. 6A). On the other hand, LPS/INFγ, but not IL4/IL13, increased NO production (open columns at Fig. 6B). By comparison to unstimulated WT cells, arginase activity was spontaneously increased by ∼2-fold in *Aqp1* ^*-/-*^ macrophages, to a similar level as induced by IL4/IL13 in WT cells (Fig. 6A, compare 2d and 5d columns). In *Aqp1* ^*-/-*^ macrophages stimulated by IL4/IL13, arginase activity increased by 4-fold, indicating an additive effect of AQP1 ablation and IL4/IL13 (Fig. 6A, 6D column). In contrast, NO level was only moderately increased (∼2-fold) by AQP1 ablation, as compared to the ∼8-fold increase induced by LPS/INFγ (Fig. 6B). The stimulation of arginase activity by AQP1 ablation to a similar level as upon IL4/IL13 further supports the hypothesis that AQP1 deficiency mimics M2 differentiation.

### AQP1 ablation paradoxically decreases migration of M1 macrophages both *in vitro* and *in vivo*

**Fig 7:**
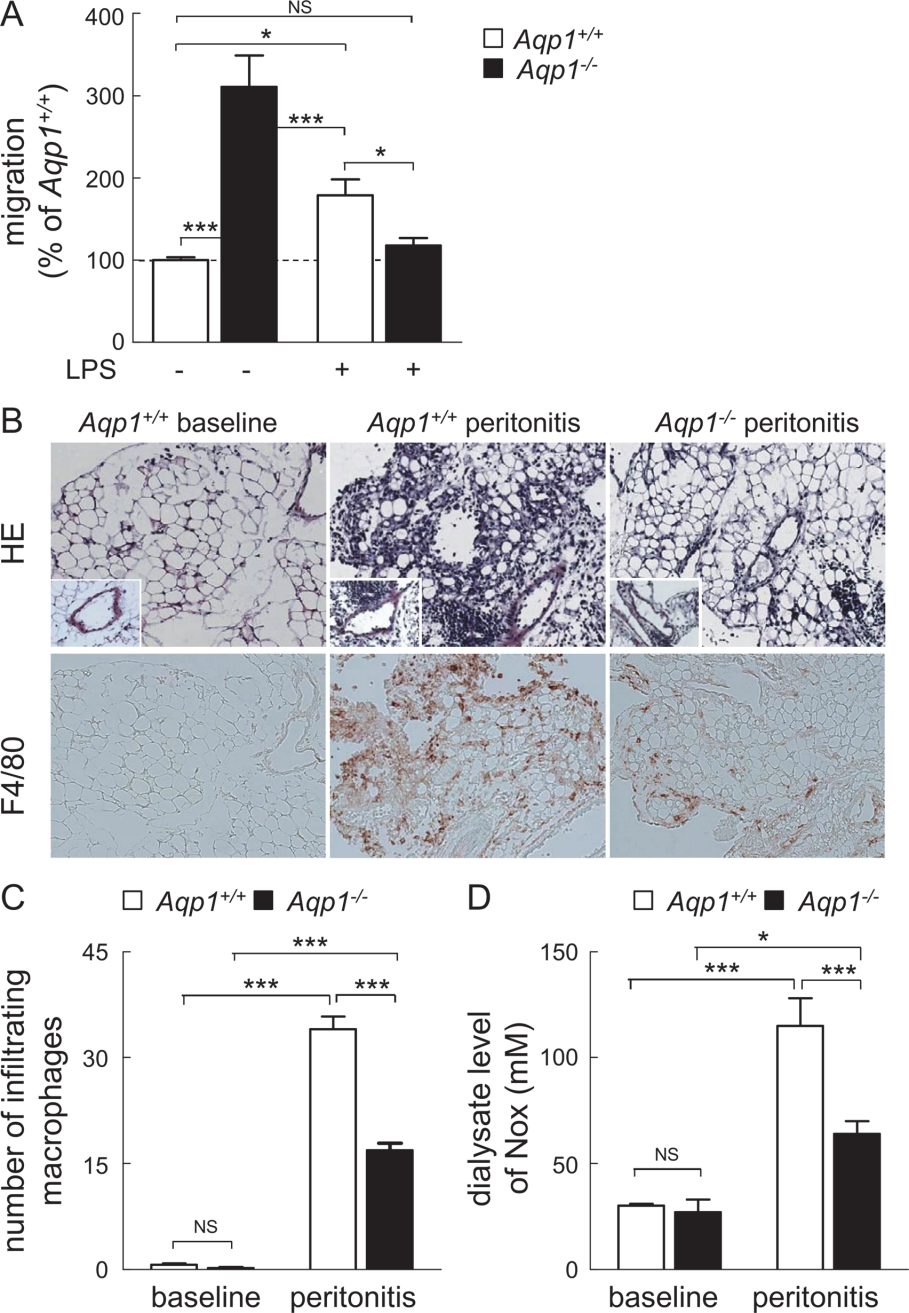
AQP1 ablation decreases macrophage migration upon LPS stimulation and in an inflammatory infiltrate. (**A) *In vitro* wound healing assay upon LPS stimulation.** Migration of *Aqp1* ^*+/+*^ (open bars) or *Aqp1* ^*-/-*^ (filled bars) macrophages was tested as in Fig. 1 in medium with serum alone (undifferentiated M0 phenotype) or supplemented by LPS (M1 phenotype). Values are expressed by reference to *Aqp1* ^*+/+*^ macrophages maintained in serum alone and are means±SEM of 4–6 experiments with 3 dishes each. **(B-D) *In vivo* peritonitis model.** The morphology (hematoxylin-eosin [HE] staining) and the presence of infiltrating macrophages (immunolabeling for F4/80) in the visceral peritoneum were assessed in *Aqp1* ^*+/+*^ and *Aqp1* ^*-/-*^ mice at baseline and 7 days after generation of an acute peritonitis (B). The peritoneal infiltrate as well as the number of infiltrating macrophages are greatly attenuated in *Aqp1* ^*-/-*^ *vs Aqp1* ^*+/+*^ mice, as confirmed by the quantification of infiltrating macrophages (C) and dialysate level of NOx (D). Original magnification, x150 (inset, x300). Representative of 4 trios of mice. NS, not significant; *, p<0.05; ***, p<0.001.

We thus concluded that AQP1 ablation constitutively enhanced cell migration by inducing a shift from M0 to M2 phenotype. However, key regulators of macrophage migration in response to infection are LPS and IFNγ. We thus examined if and how *Aqp1* ^*-/-*^ macrophage migration was responding to 1μg/ml LPS stimulation [50,52,53]. As expected [54], LPS stimulated by ∼2-fold *Aqp1* ^*+/+*^ macrophage migration in our wound healing assay (compare white bars in Fig. 7A). In comparison, migration of *Aqp1* ^*-/-*^ macrophages was decreased by ∼2.5-fold, reaching the level of WT cells in the absence of LPS (compare 1st and 4th columns at Fig. 7A). This indicated that AQP1 is necessary for the stimulation of macrophage motility by LPS. The suppression of the motility response in *Aqp1* ^*-/-*^ cells could not be attributed to silencing the LPS signalling pathway, as evidenced by comparable LPS-induced p38 MAP kinase activation, iNOS expression and IκBα phosphorylation in *Aqp1* ^*+/+*^ and *Aqp1* ^*-/-*^ cells (S5 Fig.).

The paradoxical suppression of *Aqp1* ^*-/-*^ macrophage migration in response to LPS prompted us to evaluate macrophage infiltration *in vivo*, for which we used a well-established mouse model of acute bacterial peritonitis [40]. As expected, acute peritonitis in *Aqp1* ^*+/+*^ mice was reflected by a massive, submesothelial infiltrate of mononuclear cells including macrophages (F4/80 staining). The peritoneal infiltrate was strongly attenuated in *Aqp1* ^*-/-*^ mice, with a 2-fold decreased number of infiltrating macrophages (Fig. 7B,C). These changes were mirrored by changes in dialysate levels of NOx: acute peritonitis in *Aqp1* ^*+/+*^ mice induced a 4-fold increase in NOx levels, which was decreased to ∼2-fold in *Aqp1* ^*-/-*^ mice (Fig. 7D). The decreased infiltration of *Aqp1* ^*-/-*^ macrophages in the peritonitis model was in full agreement with the abrogation of LPS response *in vitro* (Fig. 7A). We concluded that AQP1 ablation decreased macrophage migration in pro-inflammatory contexts *in vitro* and *in vivo*, which could not be explained by intrinsic refractoriness to LPS signalling.

### AQP1 ablation oppositely affects phagocytosis, depending on stimulation or not by LPS

Taken together, our data indicate that AQP1 ablation oppositely affected macrophage migration depending on stimulation or not by LPS. Since macrophage phagocytic properties also depend on phenotype polarization [26,55–57], we then evaluated phagocytosis capacity of WT and *Aqp1* ^*-/-*^ macrophages, either unstimulated (M0) or stimulated by LPS (M1). In non-stimulated macrophages (M0), AQP1 ablation increased phagocytosis of 1μm-latex beads by ∼60%. In contrast, upon M1 orientation by LPS, phagocytosis of *Aqp1* ^*-/-*^ macrophages was reduced by ∼60%, as compared to WT macrophages (S6 Fig.). These data indicate that, like for migration, AQP1 ablation also oppositely regulated macrophage phagocytosis, depending on stimulation or not by LPS.

## Discussion

### Overview

The water channel AQP1 is classically viewed as a migration facilitator in many cell types, including epithelial and endothelial cells. Here we investigated the role of AQP1 in macrophage motility in relation with phenotype polarization, using peritoneal macrophages isolated from *Aqp1* deficient mice, either unstimulated or stimulated to orientate towards M1 or M2 phenotypes. In non-stimulated macrophages, AQP1 ablation increased migration by constitutively inducing cell elongation, membrane polarization and lamellipodia formation at the leading edge via the Src/PI3K/Rac pathway. These features resembled in all tested aspects to WT macrophage transition from M0 to M2 state. We demonstrate an opposite impact of AQP1 ablation on macrophage migration, depending on absence (promotion) or stimulation by LPS (repression). We speculate that AQP1 down-regulation in macrophages may consolidate their orientation into tissue remodelling, healing and repair (M2).

### AQP1 ablation promotes macrophage migration by mimicking a M0-to-M2 switch

In absence of external stimulus, the spontaneous migration of *Aqp1* ^*-/-*^ macrophages was strongly increased in comparison to WT cells, as shown by wound healing, *de novo* colonization of IBIDI plastic chambers and Transwell migration assay, and reproduced by acute treatment of WT macrophages with the AQP blocker HgCl_2_. Accelerated migration could not be explained by an AQP7 compensatory increase. This indicates that, in contrast to other cell types studied so far, AQP1 is not essential for migration of non-stimulated macrophages. Our data support the view that the blocking effect of AQP1 on migration is specific to resting non-stimulated macrophages, which are particular for several reasons: (i) they exhibit a poorly-organized actin cytoskeleton, in contrast to epithelial and endothelial cells which elaborate stable cytoskeleton and adhesions [58]; (ii) they have higher surface tension and bending modulus as compared to many other cells such as astrocytes, glioblastoma cells, neurons or neutrophils [59,60]; and (iii) they are able to switch phenotype in response to environmental cues (M1 and M2), exhibiting different features with respect to migration, cell shape and cytoskeletal organization [26–28,61].

Since *Aqp1* ^*-/-*^ macrophages were more motile, elongated and formed lamellipodia, all characteristics of M2 macrophages [26,27,61], we tested the hypothesis that AQP1 ablation mimics switching to M2 phenotype, which is induced in WT macrophages by IL4/IL13. Four additional lines of evidence support this hypothesis. First, AQP1 ablation induced recruitment of the M2 macrophage marker CD206 [25] to the plasma membrane in extensions concentrating F4/80, as in WT cells stimulated by IL4/IL13. Second, like IL4/IL13, AQP1 ablation constitutively induced lamellipodia, in contrast to LPS which induced filopodia. Third, *Aqp1* ^*-/-*^ macrophages spontaneously increased by ∼2-fold arginase activity (M2 marker), to the same extent as upon treatment with IL4/IL13 in WT macrophages. Fourth, *Aqp1* ^*-/-*^ macrophages were still able to respond to IL4/IL13, with further increase of arginase activity. Our results are in agreement with a recent paper demonstrating that macrophage elongation can induce phenotype polarization towards M2 independently of soluble extracellular factors and can enhance the effect of M2-inducing cytokines [27]. The overall implication is that, in non-stimulated macrophages, AQP1 represses migration and switch to M2 phenotype.

To the best of our knowledge, this is the first report exploring the role of AQP1 in macrophage migration and phagocytosis. A previous study has shown that AQP3 favours macrophage migration and phagocytosis, even without stimulation by LPS [2]. The apparent discrepancy between the latter and our study might be explained by the different shape of macrophages, *Aqp3* ^*-/-*^ macrophages remaining round in contrast to elongated *Aqp1* ^*-/-*^ macrophages. Moreover, in addition to water transport, AQP3 also transports glycerol, an important intermediate of energy metabolism. Reduced ATP contents observed in *Aqp3* ^*-/-*^ macrophages may contribute to the impaired macrophage migration [2].

### AQP1 ablation instead impairs M1 macrophage migration, as in other cells

Whereas AQP1 ablation promoted the migration and phagocytosis of non-stimulated macrophages, we instead found impaired migration and phagocytosis in response to LPS, despite preserved LPS signalling. Several lines of evidences indicated that the inhibitory effect of LPS on macrophage migration and phagocytosis cannot be explained by toxicity: (i) in WT macrophages, LPS did not inhibit but instead stimulated migration and phagocytosis; (ii) in *Aqp1* ^*-/-*^ cells, LPS signalling was preserved; and (iii) in *Aqp7* ^*-/-*^ macrophages, LPS did not inhibit but instead slightly increased migration. Moreover, we observed a reduction of macrophage infiltration in an *in vivo* model of peritonitis. This indicates that AQP1 is instrumental in the machinery supporting increased migration and phagocytosis of M1 macrophages. Stimulation of migration by AQP1 was already described in a large number of cells, including proximal tubular cells [4], aortic endothelial cells [3], CHO cells [3] and human melanoma cells [19]. In these studies, AQP1 becomes generally polarized to the front of cells and is associated with increased turnover of membrane protrusions. Two other aquaporins are known to facilitate migration, i.e. the water channel AQP4 in astrocytes [3] and the aquaglyceroporin AQP3 in keratinocytes, corneal epithelial cells and fibroblasts [5]. How these various aquaporins facilitate cytosol forward displacement deserves to be further addressed.

### Possible mechanisms of the opposite regulation of motility by AQP1 in resting and LPS-activated macrophages

We will now discuss mechanistic information gained from the opposite effect of AQP1 ablation on macrophage migration, depending on absence or presence of LPS stimulation. Individual (as opposed to collective) migration generally involves membrane protrusions at the leading edge with retraction of the rear edge. Cell migration also implies the activity of ion channels and transporters which participate in the regulation of cell volume and actin filaments. The current model suggests that actin remodeling and ion uptake at the tip of lamellipodia creates a local osmotic gradient which drives AQP-mediated water influx, increasing the local cytosolic pressure, supporting membrane protrusions and creating space for actin polymerization that promotes migration [17,19].

The above model can however not explain the opposite effect of AQP1 ablation in non-stimulated *vs* LPS-stimulated macrophages. Hence, one can ask how it is possible for a water channel to block cell migration. We suggest that the inhibitory effect of AQP1 on migration is specific to cells exhibiting high elastic constants, such as macrophages. In their resting state, these cells indeed exhibit higher surface tension and bending modulus as compared to LPS-activated macrophages and many other cell types, such as astrocytes, glioblastoma cells, neurons or neutrophils [59,60]. Of interest, increase of membrane tension is known to close the constitutively open human AQP1 channel [15] and to inhibit localized Rac1 signaling [62], a crucial step to drive downstream effectors leading to cytoskeletal rearrangement, focal adhesion complex formation and lamellipodia extensions.

Thus, in WT M0 macrophages, high surface tension would close AQP1 channel [15] and impair Rac1 activation [62] and formation of cellular extensions, thus preventing migration. In contrast, stimulation of WT macrophages by LPS induced cytoskeleton remodelling with filopodia formation together with surface tension and bending modulus decreases as shown by [59], maintaining AQP1 open. Upon AQP1 ablation, a M0-to-M2 switch together with cell elongation, activation of the PI3K/Src/Rac signalling pathway and constitutive lamellipodia formation were observed, all features consistent with increased migration. It remains to be directly tested whether these features can be explained by a decrease of surface tension and whether such modification could in turn increase localized Rac1 signaling. Upon addition of LPS to these *Aqp1* ^*-/-*^ macrophages, migration was strongly decreased, suggesting that constitutive switch of M0-to-M2 upon AQP1 ablation prevents macrophages from M1 polarization by inflammatory stimuli. Accordingly, it has been shown that macrophage elongation, which can induce polarization towards M2 phenotype independently of soluble extracellular factors, inhibits the effect of M1-inducing cytokines [27].

### Implications of AQP1 in macrophage immunity

The conclusion that AQP1 ablation stimulates macrophage migration by a M0-to-M2 transition and that M2 differentiation will enhance the effect of IL4/IL13 further suggests that down-regulation of AQP1 in macrophages could help to orientate macrophages towards tissue remodelling, healing and repair [22,24,63]. Conversely, AQP1 ablation prevents adaptation to M1 phenotype by LPS. Accordingly, AQP1 ablation in macrophage is associated with a strong reduction of IL1β release and neutrophilic inflammation [44] as confirmed by our *in vivo* data showing a major effect during bacterial infection. These observations support that functional AQP1 could play a role in supporting macrophage movement to sites of infection (M1), while repressing orientation towards M2 phenotype.

## Supporting Information

S1 Fig Validation of freshly isolated *Aqp1* ^*-/-*^ peritoneal macrophages. **(A) Similar yields of peritoneal macrophages obtained by peritoneal lavage of *Aqp1*** ^***+/+***^ **and *Aqp1*** ^***-/-***^ **mice.** All cells collected by peritoneal lavage from *Aqp1* ^*+/+*^ and *Aqp1* ^-/-^ mice were immunolabelled for F4/80 and CD11b and analyzed by FACS. **(B) *Aqp1*** ^***+/+***^ **and *Aqp1*** ^***-/-***^ **macrophages are >99% pure**. Macrophages in peritoneal collections were identified by confocal microscopy based on phagocytosis of 1-μm latex beads and immunolabelling for F4/80, by reference to F-actin. Briefly, cells were seeded in serum-containing medium. After 24h, they were allowed to phagocytose latex beads (blue), fixed/permeabilized and (immuno)labelled for F4/80 (red) and F-actin (phalloidin; green). Counting of fibroblasts *vs* macrophages was performed on 417 *Aqp1* ^*+/+*^ and 410 *Aqp1* ^*-/-*^ cells from 4 independent experiments each. Insets show typical macrophages. Arrowhead points to a rare fibroblast (< 1% of the cell collection). Scale bars, 20μm. **(C) Validation of AQP1 antibodies and *Aqp1*** ^***-/-***^ **cells.** A clear band at ∼25kDa is observed in four isolates of *Aqp1* ^*+/+*^ macrophages (left) but not in *Aqp1* ^*-/-*^ macrophages (right); β-actin as internal loading control.
(TIFF)

S2 Fig AQP1 ablation increases spontaneous macrophage motility in medium without serum and supplements, as shown in three different assays (complementary to Fig. 1). **(A) Wound healing assay.** Similar assay as described at Fig. 1, but in the absence of serum. **(B) *De novo* colonization (IBIDI chamber).** *Aqp1* ^*+/+*^ and *Aqp1* ^*-/-*^ cells maintained in serum-free medium were allowed to migrate in the cell-free area of IBIDI chamber for 24h, as described at Fig. 3D. Number of cells that had colonized the fresh area was reported to the number of macrophages introduced in the well. **(C) Transwell migration assay.** *Aqp1* ^*+/+*^ and *Aqp1* ^*-/-*^ cells were seeded on Transwell inserts and allowed to migrate towards lower chambers in serum-free medium. 24h later, cells were stained and those that had not migrated to the lower chamber were wiped with a cotton-tipped swab after which cells that had migrated were counted. Data from all three assays are expressed by reference to *Aqp1* ^*+/+*^ macrophages and are means±SEM of 3–4 experiments with 1–3 dishes each, except at panel B (1 experiment). *, p<0.05; **, p<0.01.
(TIFF)

S3 Fig AQP7 ablation does not appreciably affect macrophage migration in wound healing. *Aqp7* ^*+/+*^ (open bars) and *Aqp7* ^*-/-*^ macrophages (black bars) were scraped and migration was tested by wound healing assay in medium with serum alone (left) or supplemented with LPS (right) as at Fig. 1. Data are from two independent experiments and are expressed by reference to migration of *Aqp7* ^*+/+*^ macrophages in serum.
(TIFF)

S4 Fig AQP1 ablation spontaneously increases macrophage elongation: general views (complementary to Fig. 3A,B). *Aqp1* ^*+/+*^ and *Aqp1* ^-/-^ macrophages were maintained for 24h in serum-containing medium, then fixed/permeabilized and labelled for F-actin by phalloidin. Scale bars, 10μm. For quantification of macrophage elongation, see Fig. 3B.
(TIFF)

S5 Fig AQP1 ablation does not affect LPS signalling. *Aqp1* ^*+/+*^ and-^/-^ macrophages were maintained for 24h in serum-containing medium supplemented or not with LPS. Total lysates were assessed for phosphorylation of p38 MAP kinase (A), for expression of iNOS (B) and for phosphorylation and proteasome-mediated degradation of the NF-κB inhibitor protein IκBα (C). GAPDH (A) and β-actin (B,C) were used as internal loading controls. Representative blots of 2 experiments at each panel are shown.
(TIFF)

S6 Fig AQP1 ablation differentially affects phagocytosis of latex beads, depending on stimulation or not by LPS. *Aqp1* ^*+/+*^ and *Aqp1* ^*-/-*^ macrophages were maintained in serum-containing medium without supplements (to keep macrophages in undifferentiated state; upper row), or with LPS (to orient towards M1 phenotype; lower row). Cells were then incubated for 1h with 1μm-latex beads (green) and fixed/permeabilized with formaldehyde/saponine, then labelled for Alexa 568-phalloidin (red). **(A) Representative images.** Representative of three independent experiments. Scale bars, 5μm. **(B) Quantification of latex beads phagocytosis.** Values are means±SEM of two independent experiments with two coverslips each (14 to 17 macrophages were counted per condition) and are expressed by reference to *Aqp1* ^*+/+*^ macrophages without LPS. NS, not significantly different; ***, p<0.001.
(TIFF)

S1 Table Primers used in real-time RT-PCR analyses. The primers were designed using Beacon Design 2.0 (Premier Biosoft International, Palo Alto, CA). The efficiency of the reactions was calculated as explained in [30].
(TIFF)

S2 Table Expression levels of aquaporins mRNA in *Aqp1* ^-/-^ *vs* WT macrophages. The threshold Ct values were obtained from seven different WT mice. ND, transcript not detected or Ct> 34 cycles. NS, not significant; *, p<0.05.
(TIFF)
