# Supplementary figures and images for: Regulation of Macrophage Motility by the Water Channel Aquaporin-1: Crucial Role of M0/M2 Phenotype Switch
Source: PLoS One. 2015 Feb 26;10(2):e0117398. doi: 10.1371/journal.pone.0117398 (PMC4342038; doi:10.1371/journal.pone.0117398)

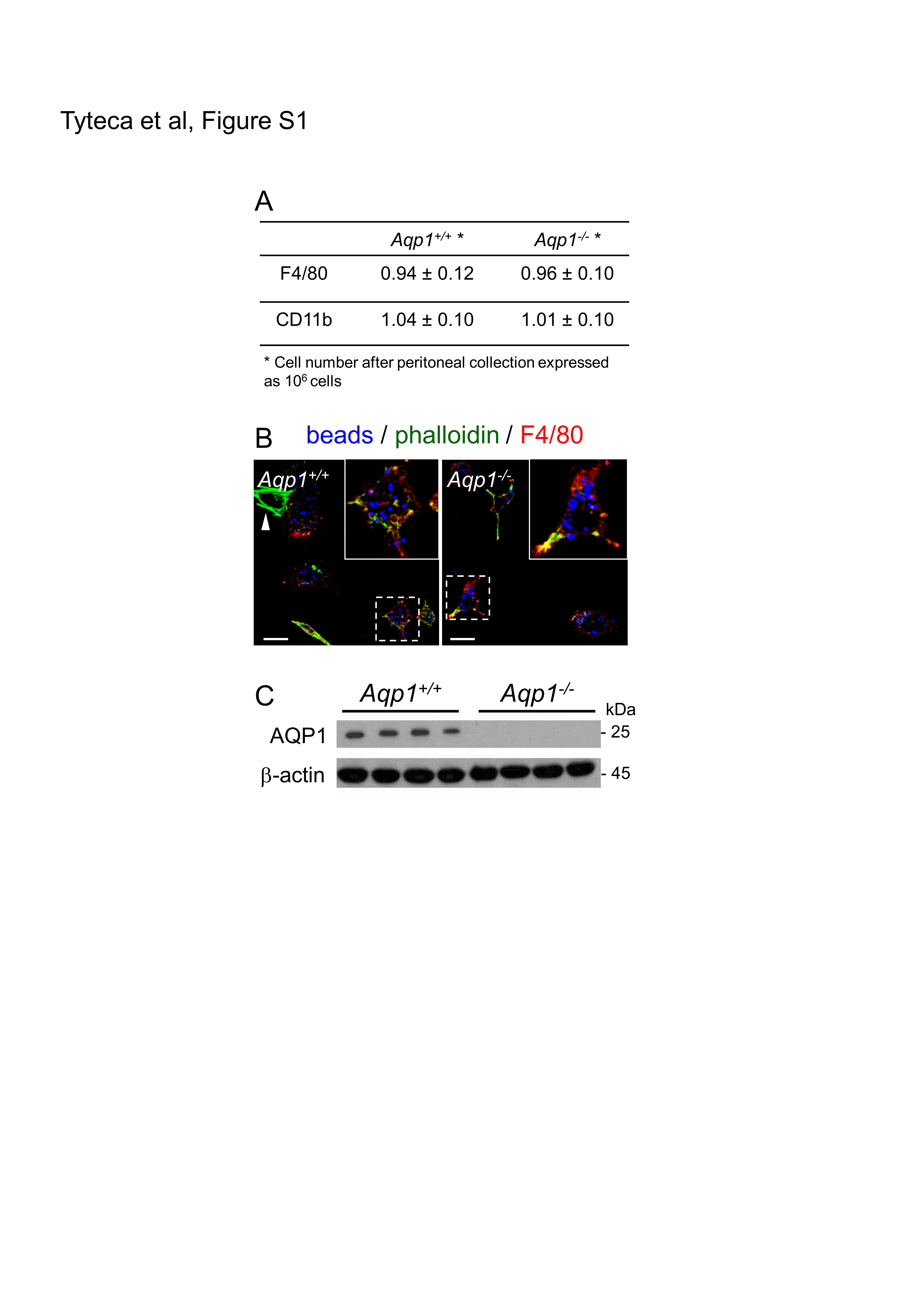

Supplement: S1 Fig — (A) Similar yields of peritoneal macrophages obtained by peritoneal lavage of Aqp1 +/+ and Aqp1 -/- mice. All cells collected by peritoneal lavage from Aqp1 +/+ and Aqp1 -/- mice were immunolabelled for F4/80 and CD11b and analyzed by FACS. (B) Aqp1 +/+ and Aqp1 -/- macrophages are >99% pure. Macrophages in peritoneal collections were identified by confocal microscopy based on phagocytosis of 1-μm latex beads and immunolabelling for F4/80, by reference to F-actin. Briefly, cells were seeded in serum-containing medium. After 24h, they were allowed to phagocytose latex beads (blue), fixed/permeabilized and (immuno)labelled for F4/80 (red) and F-actin (phalloidin; green). Counting of fibroblasts vs macrophages was performed on 417 Aqp1 +/+ and 410 Aqp1 -/- cells from 4 independent experiments each. Insets show typical macrophages. Arrowhead points to a rare fibroblast (< 1% of the cell collection). Scale bars, 20μm. (C) Validation of AQP1 antibodies and Aqp1 -/- cells. A clear band at ∼25kDa is observed in four isolates of Aqp1 +/+ macrophages (left) but not in Aqp1 -/- macrophages (right); β-actin as internal loading control. (TIFF) [file pone.0117398.s001.tiff]

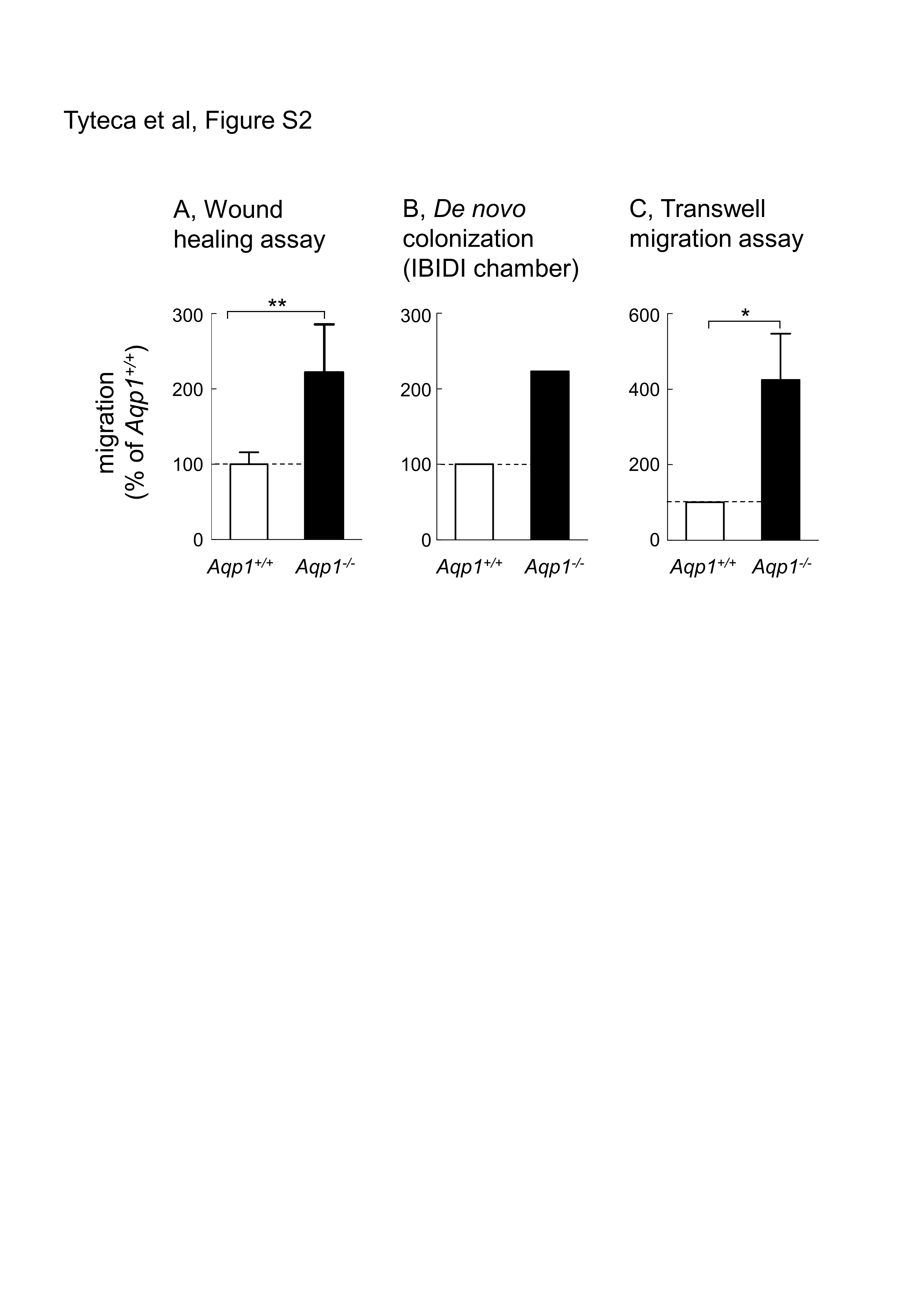

Supplement: S2 Fig — (A) Wound healing assay. Similar assay as described at Fig. 1, but in the absence of serum. (B) De novo colonization (IBIDI chamber). Aqp1 +/+ and Aqp1 -/- cells maintained in serum-free medium were allowed to migrate in the cell-free area of IBIDI chamber for 24h, as described at Fig. 3D. Number of cells that had colonized the fresh area was reported to the number of macrophages introduced in the well. (C) Transwell migration assay. Aqp1 +/+ and Aqp1 -/- cells were seeded on Transwell inserts and allowed to migrate towards lower chambers in serum-free medium. 24h later, cells were stained and those that had not migrated to the lower chamber were wiped with a cotton-tipped swab after which cells that had migrated were counted. Data from all three assays are expressed by reference to Aqp1 +/+ macrophages and are means±SEM of 3–4 experiments with 1–3 dishes each, except at panel B (1 experiment). *, p<0.05; **, p<0.01. (TIFF) [file pone.0117398.s002.tiff]

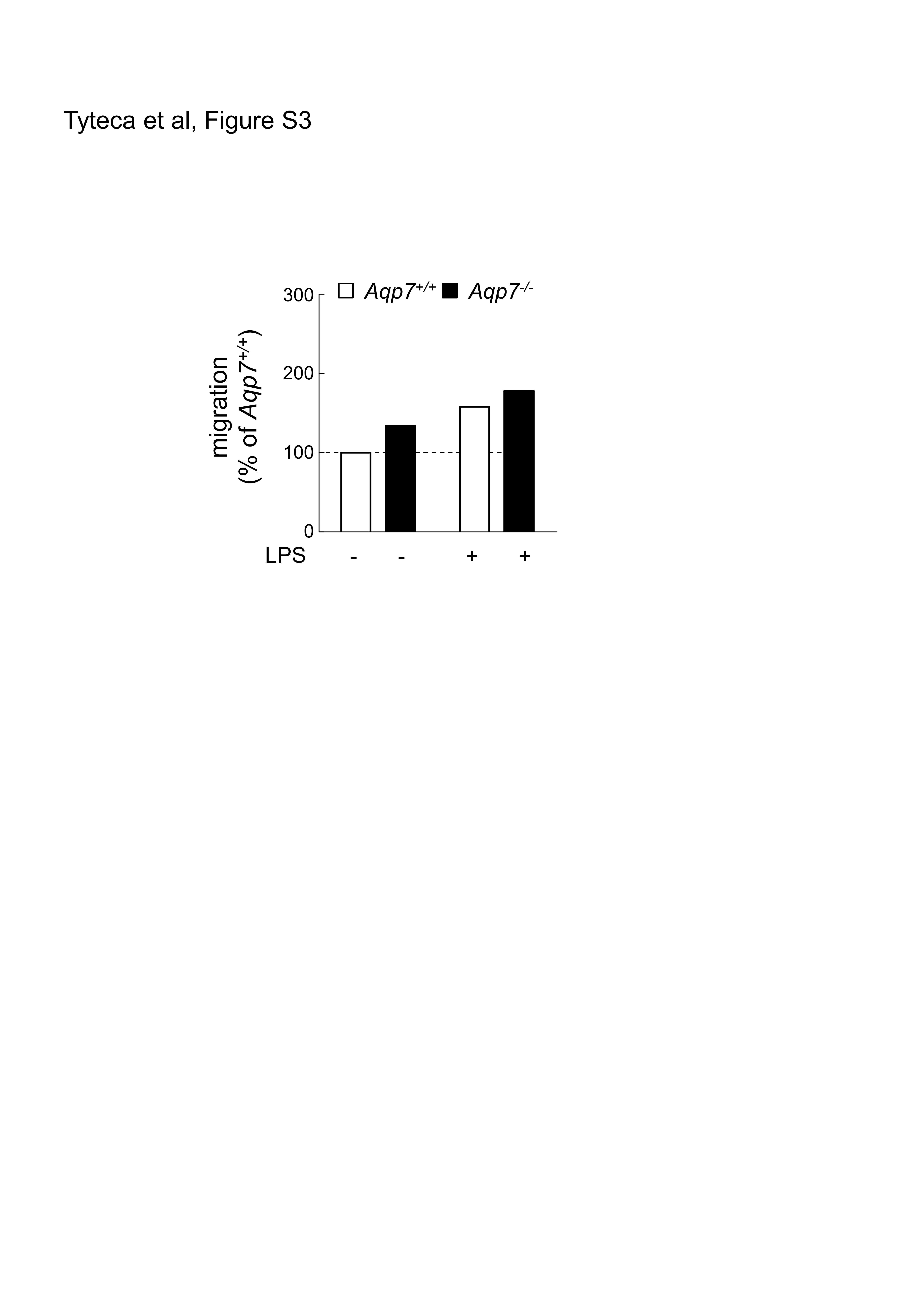

Supplement: S3 Fig — Aqp7 +/+ (open bars) and Aqp7 -/- macrophages (black bars) were scraped and migration was tested by wound healing assay in medium with serum alone (left) or supplemented with LPS (right) as at Fig. 1. Data are from two independent experiments and are expressed by reference to migration of Aqp7 +/+ macrophages in serum. (TIFF) [file pone.0117398.s003.tiff]

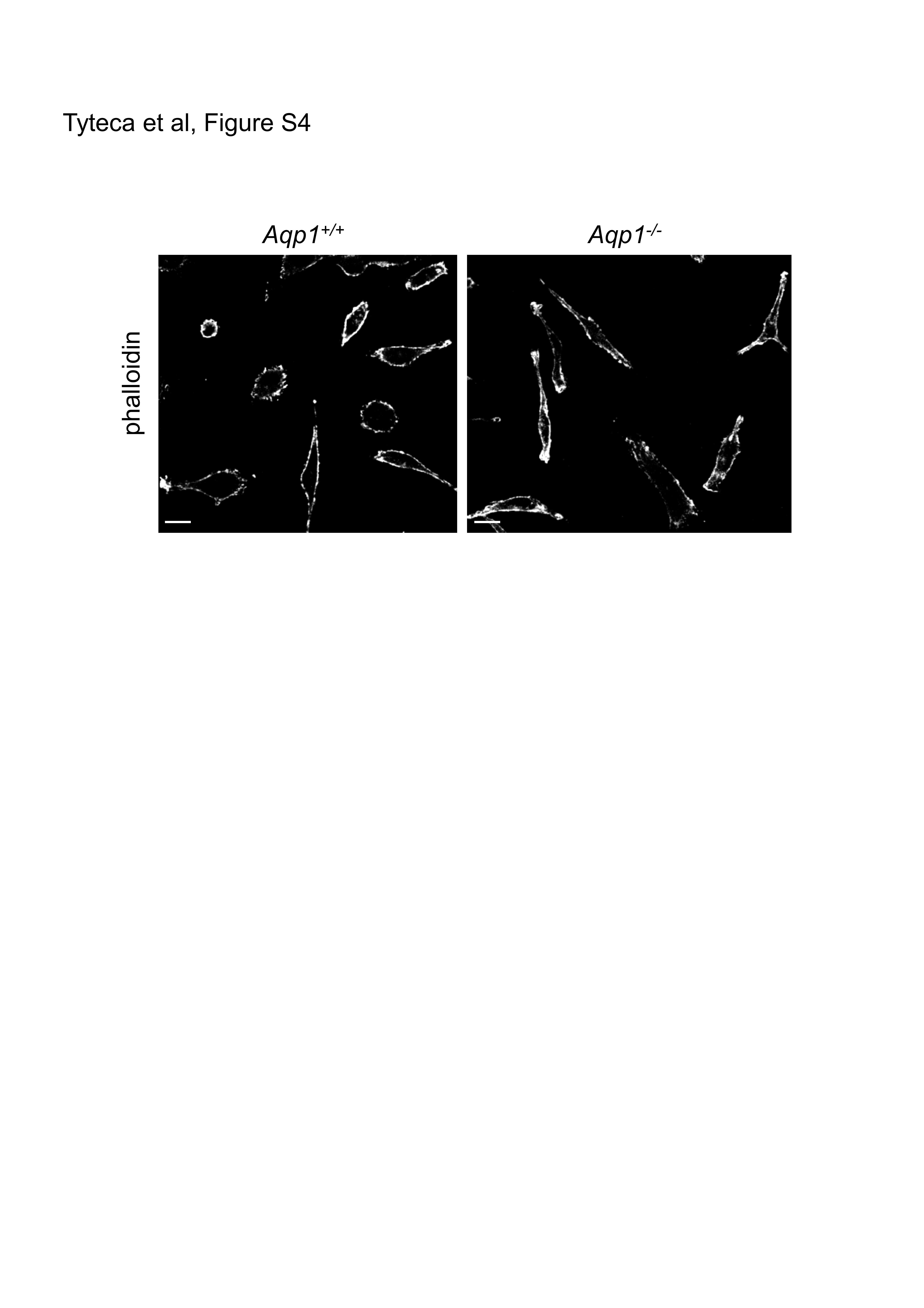

Supplement: S4 Fig — Aqp1 +/+ and Aqp1 -/- macrophages were maintained for 24h in serum-containing medium, then fixed/permeabilized and labelled for F-actin by phalloidin. Scale bars, 10μm. For quantification of macrophage elongation, see Fig. 3B. (TIFF) [file pone.0117398.s004.tiff]

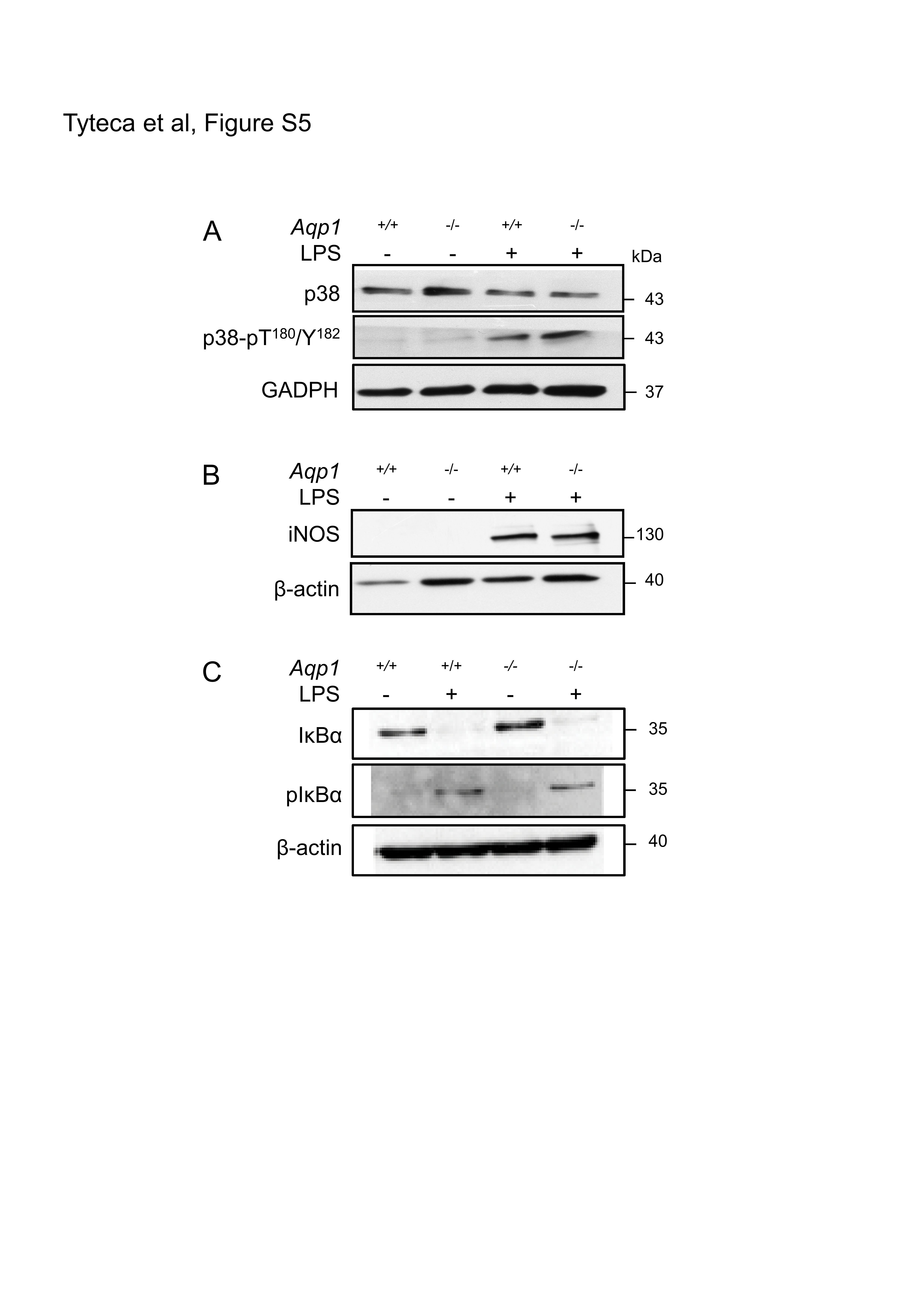

Supplement: S5 Fig — Aqp1 +/+ and-/- macrophages were maintained for 24h in serum-containing medium supplemented or not with LPS. Total lysates were assessed for phosphorylation of p38 MAP kinase (A), for expression of iNOS (B) and for phosphorylation and proteasome-mediated degradation of the NF-κB inhibitor protein IκBα (C). GAPDH (A) and β-actin (B,C) were used as internal loading controls. Representative blots of 2 experiments at each panel are shown. (TIFF) [file pone.0117398.s005.tiff]

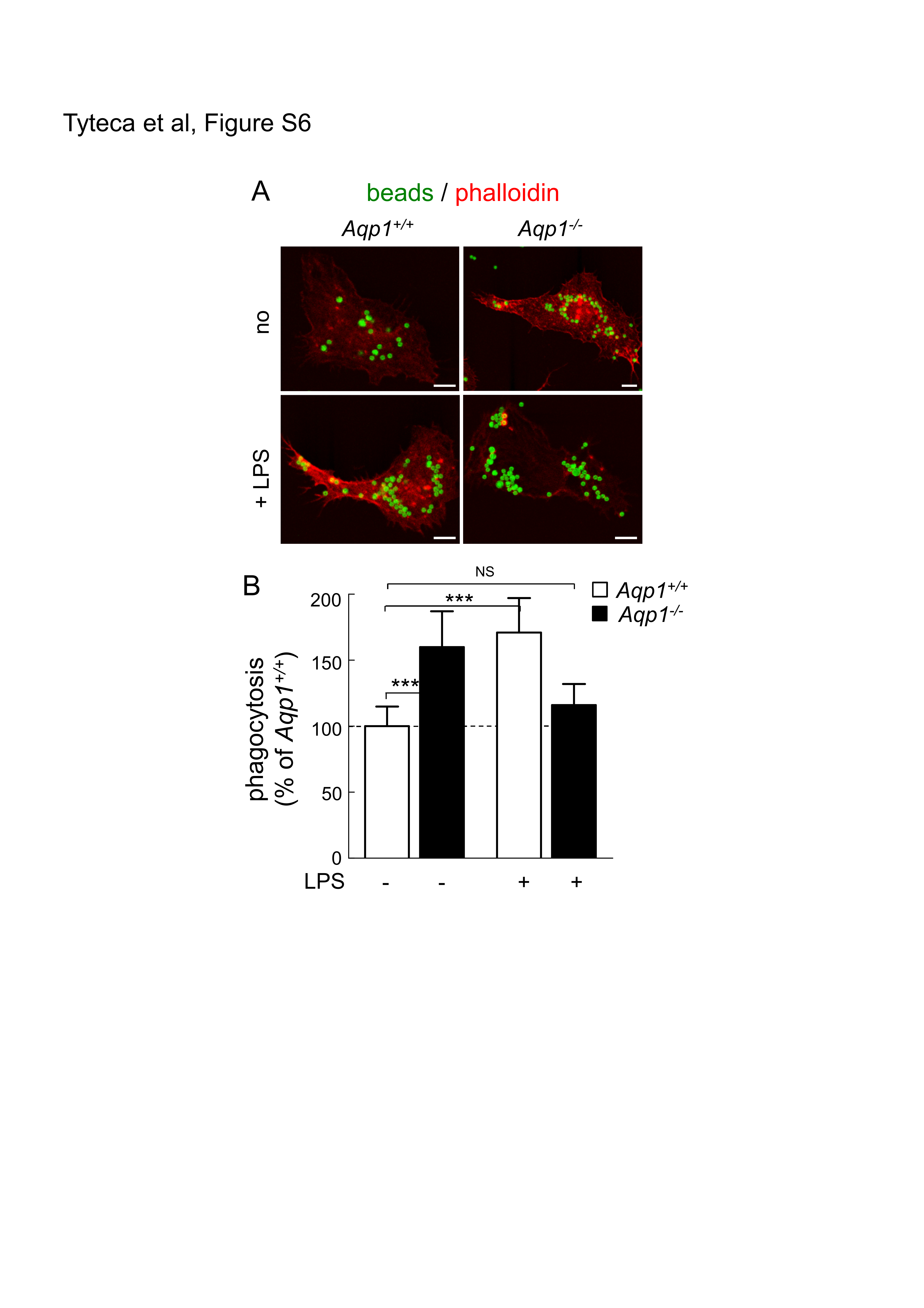

Supplement: S6 Fig — Aqp1 +/+ and Aqp1 -/- macrophages were maintained in serum-containing medium without supplements (to keep macrophages in undifferentiated state; upper row), or with LPS (to orient towards M1 phenotype; lower row). Cells were then incubated for 1h with 1μm-latex beads (green) and fixed/permeabilized with formaldehyde/saponine, then labelled for Alexa 568-phalloidin (red). (A) Representative images. Representative of three independent experiments. Scale bars, 5μm. (B) Quantification of latex beads phagocytosis. Values are means±SEM of two independent experiments with two coverslips each (14 to 17 macrophages were counted per condition) and are expressed by reference to Aqp1 +/+ macrophages without LPS. NS, not significantly different; ***, p<0.001. (TIFF) [file pone.0117398.s006.tiff]

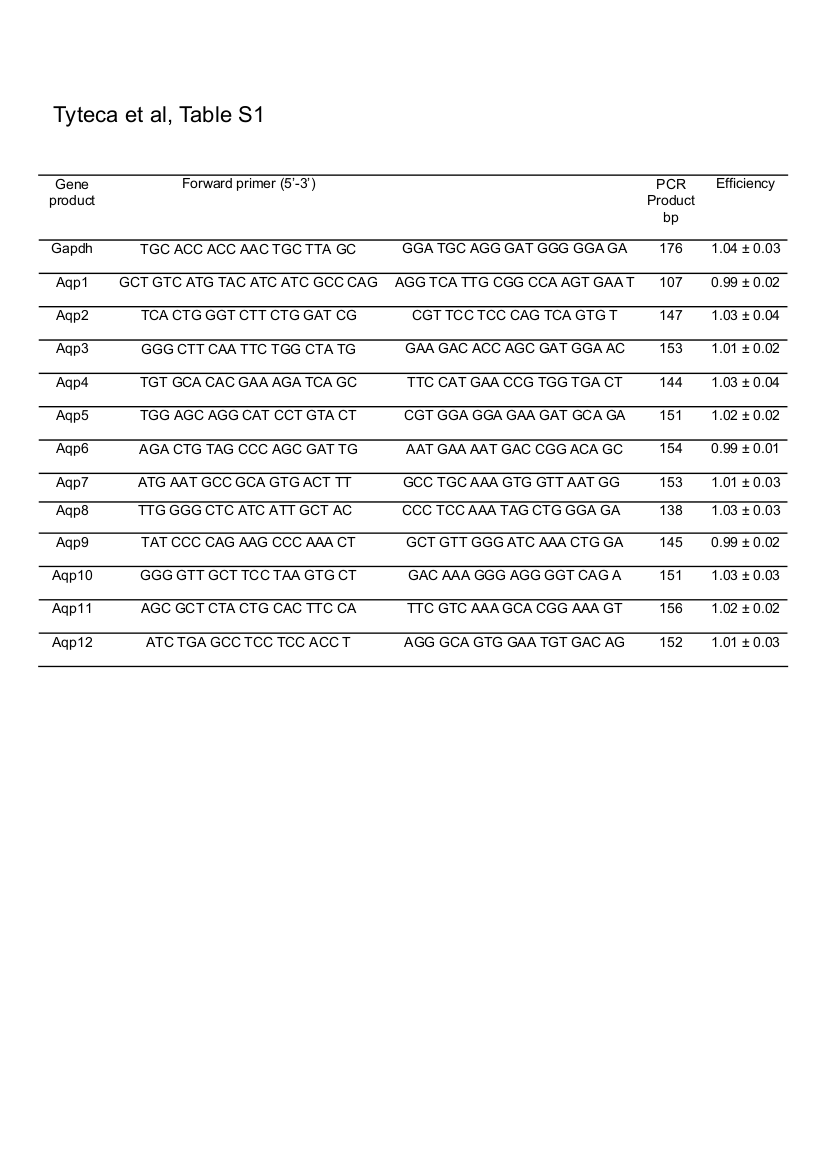

Supplement: S1 Table — The primers were designed using Beacon Design 2.0 (Premier Biosoft International, Palo Alto, CA). The efficiency of the reactions was calculated as explained in [30]. (TIFF) [file pone.0117398.s007.tiff]

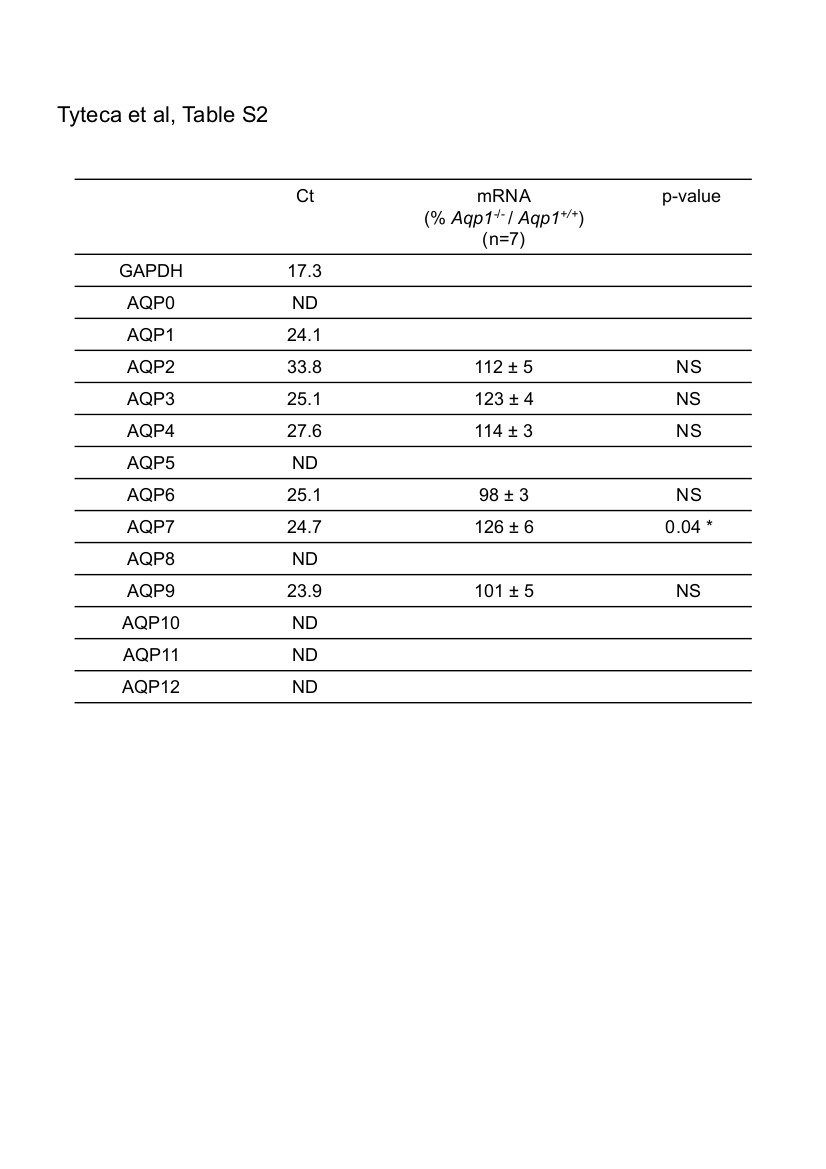

Supplement: S2 Table — The threshold Ct values were obtained from seven different WT mice. ND, transcript not detected or Ct> 34 cycles. NS, not significant; *, p<0.05. (TIFF) [file pone.0117398.s008.tiff]
